# Evolutionary redesign of the Atlantic cod (*Gadus morhua* L.) Toll-like receptor repertoire by gene losses and expansions

**DOI:** 10.1038/srep25211

**Published:** 2016-04-29

**Authors:** Monica H. Solbakken, Ole K. Tørresen, Alexander J. Nederbragt, Marit Seppola, Tone F. Gregers, Kjetill S. Jakobsen, Sissel Jentoft

**Affiliations:** 1 Centre for Ecological and Evolutionary Synthesis (CEES), Department of Biosciences, University of Oslo, Oslo, Norway; 2 Department of Medical Biology, The Arctic University of Norway, Tromsø, Norway; 3 Department of Biosciences, University of Oslo, Oslo, Norway; 4 Department of Natural Sciences, University of Agder, Kristiansand, Norway; 5 Research Group for Biomedical Informatics, Department of Informatics, University of Oslo, Oslo, Norway

## Abstract

Genome sequencing of the teleost Atlantic cod demonstrated loss of the Major Histocompatibility Complex (MHC) class II, an extreme gene expansion of MHC class I and gene expansions and losses in the innate pattern recognition receptor (PRR) family of Toll-like receptors (TLR). In a comparative genomic setting, using an improved version of the genome, we characterize *PRRs* in Atlantic cod with emphasis on *TLRs* demonstrating the loss of *TLR1/6*, *TLR2* and *TLR5* and expansion of *TLR7*, *TLR8*, *TLR9*, *TLR22* and *TLR25*. We find that Atlantic cod *TLR* expansions are strongly influenced by diversifying selection likely to increase the detectable ligand repertoire through neo- and subfunctionalization. Using RNAseq we find that Atlantic cod *TLRs* display likely tissue or developmental stage-specific expression patterns. In a broader perspective, a comprehensive vertebrate *TLR* phylogeny reveals that the Atlantic cod *TLR* repertoire is extreme with regards to losses and expansions compared to other teleosts. In addition we identify a substantial shift in *TLR* repertoires following the evolutionary transition from an aquatic vertebrate (fish) to a terrestrial (tetrapod) life style. Collectively, our findings provide new insight into the function and evolution of *TLRs* in Atlantic cod as well as the evolutionary history of vertebrate innate immunity.

Functional understanding of teleost immunity and its diversity is still in its infancy. Homologs of both mammalian innate and adaptive immune genes have been detected in teleost genomes, however, teleosts display greater genetic diversity as well as some functional discrepancies - for examples see references^1^,^2^,^3^. Central to innate immunity are pattern recognition receptors (PRRs) that detect pathogen associated molecular patterns (PAMPs) and initiate various features of the host’s immune system - see^4^ and references therein. One of the largest PRR families is the Toll-like receptors (TLRs). Upon ligand interaction, TLRs initiate the production of cytokines, anti-viral components and co-stimulatory molecules via the TLR signalling pathway - see^5^ and references therein. The diversity of TLR repertoires among multicellular organisms is substantial. The invertebrate TLR repertoire spans from several hundred genes in the sea urchin (*Strongylocentrotus purpuratus*) to only two genes in the ascidian *Ciona intestinalis*^6^. This is in stark contrast to the less extensive vertebrate repertoire that generally display between 10–13 TLR genes - overview in^7^,^8^,^9^.

Currently, there are ~20 known vertebrate *TLRs* (*TLR1-26*, the annotation used for individual genomes varies) where mammals display *TLR1-13* in contrast to fish which also display *TLR14*–*26*. Vertebrate *TLRs* form six families; *TLR1*, *TLR3*, *TLR4*, *TLR5*, *TLR7* and *TLR11* and individual species generally harbours at least one member from each family^8^. However, some exceptions are known such as the lack of *TLR11 -family* representatives in mammals. Teleosts display greater genetic diversity of *TLRs* but functional studies on mammalian *TLR* homologs overall report identical protein function - see^7^,^8^.

In contrast to the genetic diversity found within the innate immune system the adaptive immune system is shown to display an intra-genetic polymorphic nature, i.e. to enable adaptation of the immune response towards specific targets^10^. Large structural or functional alterations affecting acquired immunity have been perceived as less likely. During the last decade, however, several alternative immune strategies have been identified in vertebrate species - for details see^1^,^11^,^12^. Atlantic cod (*Gadus morhua*) is a particularly interesting case as genome sequencing revealed complete loss of the *MHC-II* pathway accompanied by an extreme gene expansion of *MHC-I* and gene losses and expansions within the *TLRs*^13^,^14^,^15^. By taking advantage of a new and substantially improved genome assembly combined with large scale genomic analyses we here perform a deep characterization of the major innate immune gene families in Atlantic cod, with emphasis on *TLRs*. Our phylogenetic analysis shows that the gene losses and expansions in Atlantic cod are extreme compared to other vertebrate lineages, including other teleosts. Comparative gene syntenies firmly establish the loss of *TLR1/6*, *TLR2* and *TLR5* and expansion of *TLR7*, *TLR8*, *TLR9*, *TLR22* and *TLR25*. Further, we are also able to more accurately determine *TLR* copy number, characterize *TLRs* not found in the earlier version of the genome and perform multiple selection analyses. We detect varying numbers of sites under diversifying selection within the *TLR* expansions most likely increasing the detectable ligand repertoire through neo- and subfunctionalization. Protein structure modelling and phylogenetic analysis suggest that *TLR* losses do not reduce the available genetic toolkit to detect pathogens. Furthermore, our transcriptome profiling of Atlantic cod *TLRs* show a likely tissue specific paralog usage. Finally, a comprehensive vertebrate *TLR* phylogeny demonstrates that there is a shift in *TLR* repertoires following the transition from aquatic to terrestrial life styles mirroring different selective pressures in the two environments.

## Results

### Atlantic cod PRR gene families – the deviating TLRs

We have investigated all major PRR gene families in Atlantic cod using the new and improved genome assembly (for details see method section “Genome assembly”). The *TLR* repertoire in Atlantic cod is clearly different compared to the other investigated teleosts and vertebrates. Within the collectin, pentraxin, retinoic acid-inducible (RIG) 1-like and nucleotide-binding oligomerization domain (NOD)-like families no clear differences were found – except for two genes: Atlantic cod has no evident homolog of *NOD2* and *AIM2* (Supplementary Tables 1–3). We have therefore focused on the *TLR* repertoire in the following investigations.

### Gene syntenies verify TLR gene losses and expansions

**Figure 1:**
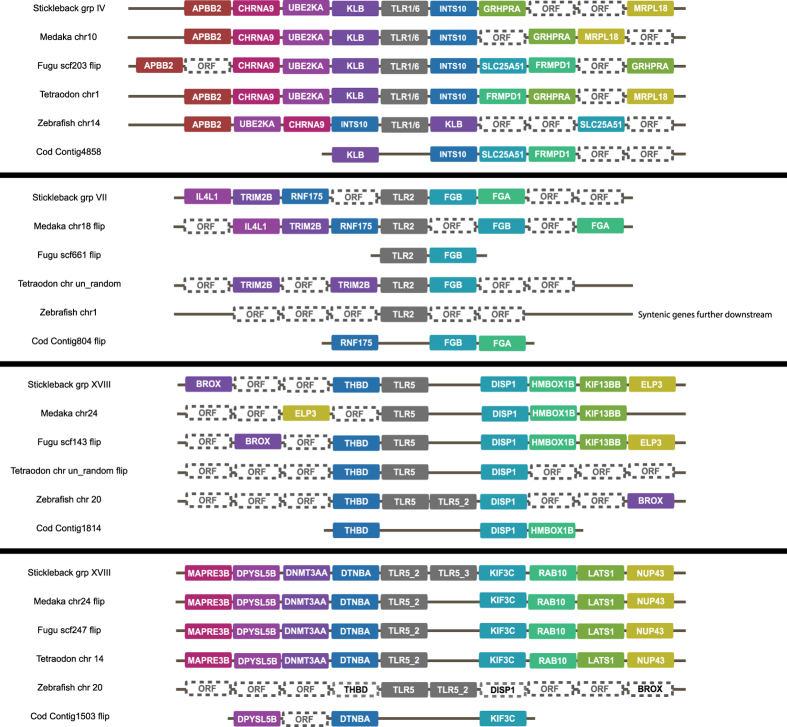
Gene synteny comparison of genomic regions in Atlantic cod towards genomic regions in stickleback, medaka, fugu, tetraodon and zebrafish containing *TLRs* not found in Atlantic cod (*TLR1/6*, *TLR2* and *TLR5*). Genes with colored boxes were found in several of the investigated species whereas white boxes designated ORF represents open reading frames which are species-specific and without certain annotation. Some genomic regions have been drawn in reversed order for visual purposes – designated “flip”. For *TLR1/6* synteny is well conserved upstream of the *TLR* where zebrafish show a local inversion. Downstream of *TLR1/6* several genes are syntenic, but the gene order varies between species and there are some species - specific open reading frames. Atlantic cod has one contig that display syntenic genes towards the other species demonstrating the loss of *TLR1/6* from its genome. For *TLR2* synteny is less conserved, however, several common genes are found. *TLR2* in zebrafish is not located to the same genomic region as in the other fish; however, the syntenic genes are located further downstream on zebrafish chromosome 1. The fugu scaffold containing *TLR2* is short and only contains one additional annotation. Atlantic cod displays three syntenic genes, but no *TLR2*, demonstrating the loss of this gene. There were two genomic regions containing *TLR5* in the investigated species. The first *TLR5* region displays limited synteny upstream but more conserved synteny downstream of *TLR5*. Zebrafish has its two *TLR5* genes tandemly organized and also seems to have a local inversion compared to the other fish. Synteny is well conserved in the second *TLR5* region with the exception of zebrafish. Atlantic cod has one additional open reading frame compared to the other species. The syntenic genes in both putative *TLR5* regions in Atlantic cod demonstrate the loss of *TLR5* from its genome.

**Figure 2:**
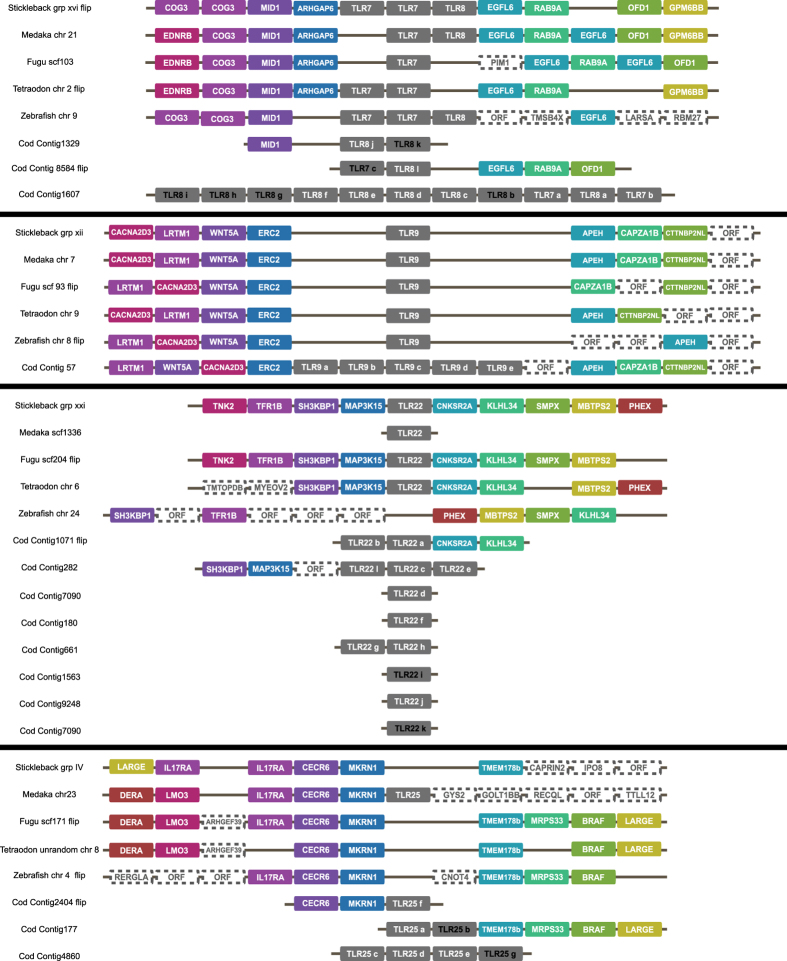
Gene synteny comparison of genomic regions in Atlantic cod towards genomic regions in stickleback, medaka, fugu, tetraodon and zebrafish containing *TLR7*, *TLR8*, *TLR9*, *TLR22* and *TLR25*. Genes with colored boxes were found in several of the investigated species whereas white boxes designated ORF represents open reading frames which are species-specific without certain annotation. Some genomic regions have been drawn in reversed order for visual purposes – designated “flip”. *TLRs* in Atlantic cod removed from further analyses due to lacking expression and/or poor sequence quality listed in Supplementary Table S1 4 are written in black. *TLR7* and *TLR8* are located to the same genomic regions in the investigated fish species. Gene synteny is well conserved, however, zebrafish displays additional open reading frames of which some have proper annotation. Stickleback, tetraodon and zebrafish have two *TLR7* whereas fugu and tetraodon lacks *TLR8*. Atlantic cod has three contigs containing both *TLR7* and *TLR8* copies interspersed. Two of these contigs have partial synteny towards the other fish species. *TLR9* is also located to genomic regions with conserved synteny. Zebrafish displays less synteny downstream of its *TLR9*. Atlantic cod has five *TLR9* copies tandemly organized on a single contig with well conserved synteny. Also *TLR22* is located to a genomic region with relatively conserved synteny among the fish species. Medaka *TLR22* is present on a scaffold with no other annotated genes present. No *TLR22* was found in zebrafish and this species has a local inversion in the predicted *TLR22* region. Atlantic cod has eight contigs with *TLR22* gene copies present where two display partial synteny and tandem organization of the *TLR22* copies. The remaining contigs are short and contains only that single gene. The predicted *TLR25* regions have relatively well conserved synteny; however, synteny is absent downstream of medaka *TLR25* and somewhat disturbed downstream in stickleback and upstream in zebrafish. *TLR25* was only found in medaka and Atlantic cod. Atlantic cod *TLR25* copies are present on three contigs of which two have partial synteny. Contigs with several *TLR25* copies display tandem organization.

We performed gene synteny analyses on all genomic regions in the assembly containing complete *TLRs* in Atlantic cod against the genomes of medaka (*Oryzias latipes*), fugu (*Takifugu rubripes*), tetraodon (*Tetraodon nigroviridis*), zebrafish (*Danio rerio*) and stickleback (*Gasterosteus aculeatus*). We found conserved gene organization up- and downstream of *TLR1/6*, *TLR2* and *TLR5* proving their absence from the Atlantic cod genome. Comparatively, each species contained some genomic reshuffling and additional open reading frames – particularly prominent in zebrafish (Fig. 1). We find that *TLR7*, *TLR8*, *TLR9*, *TLR22* and *TLR25* are expanded in Atlantic cod and that the gene copies display both tandem and non-tandem organization in numerous contigs (Fig. 2). The *TLR8* and *TLR22* expansions are the most numerous with twelve copies each. The three *TLR7* copies are interspersed among the twelve *TLR8* copies. They are present in three different contigs where two have partial gene synteny compared to the other investigated teleosts (Fig. 2). Again, zebrafish display the most deviating local genomic architecture (Fig. 2). The five copies of *TLR9* are tandemly organized on a single contig that display general conserved synteny with the other species, however with some minor gene shuffling (Fig. 2). The twelve copies of *TLR22* are found in eight contigs. Three of these contigs have tandem organization of the *TLR22* copies, but most contigs are short and only contain a single gene. In only two contigs could synteny with flanking genes be determined (Fig. 2). The *TLR22* synteny also reveals that zebrafish has lost *TLR22.* This species also harbours a local inversion involving four genes downstream of the predicted *TLR22* region and display several additional open reading frames upstream compared to the other investigated species (Fig. 2). Finally, *TLR25* consists of seven copies in Atlantic cod found in three contigs. Two of the contigs demonstrate partial synteny and contigs with several *TLR25* copies display tandem organization. Medaka was the only other species containing *TLR25* and no local synteny directly downstream of the *TLR25* genomic region was evident for this species (Fig. 2). The single copy Atlantic cod *TLRs*, *TLR3*, *TLR14*, *TLR21* and *TLR23* were also located to genomic regions displaying conserved local synteny compared to the other investigated species (data not shown).

### *TLR* expression patterns using RNAseq

**Figure 3:**
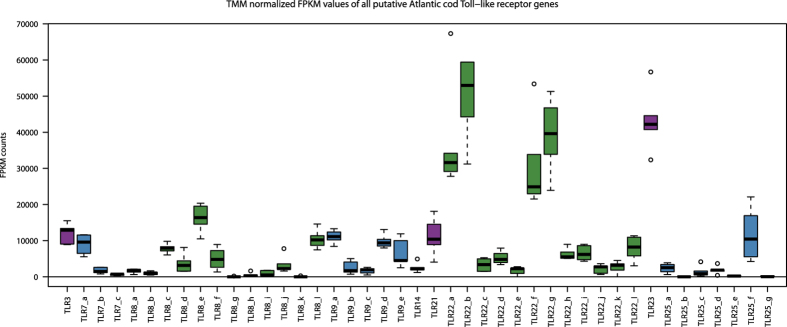
Transcriptome profiling of all Atlantic cod *TLRs*. Adapter and quality trimmed 100 bp paired-end Illumina RNAseq reads derived from the head kidney/spleen of six healthy juvenile cod were mapped towards an index of all full-length *TLRs* in Atlantic cod (S1 Table 2). The raw counts were converted to TMM normalized FPKM values and are displayed here as a box plot with average, standard deviation and outliers. The boxes have been colored for visualization purposes only. Some paralogs of *TLR7*, *TLR8* and *TLR25* have very low expression counts and the remaining *TLR* expansions display highly variable expression levels.

To investigate *TLR* expression patterns in Atlantic cod we performed RNAseq using the spleen/head kidney of healthy juvenile cod where the resulting reads were mapped towards all full-length *TLRs* found in the new Atlantic cod genome assembly. Most of the 43 full-length *TLRs* had detectable expression levels; however, four *TLRs* (two *TLR8* and two *TLR25*) had very low to no detectable expression. For the remaining *TLRs*, substantial variation in expression levels was observed (Fig. 3). The four genes with the lowest expression levels also displayed poor sequence quality resulting in protein translations containing frameshifts and stop codons possibly indicating pseudogenes. This was also the case for an additional six *TLRs*. In total 10 full-length *TLR* genes were excluded from further analysis (Supplementary Table 4).

### Endolysosomal sorting signals in Atlantic cod

**Figure 4:**
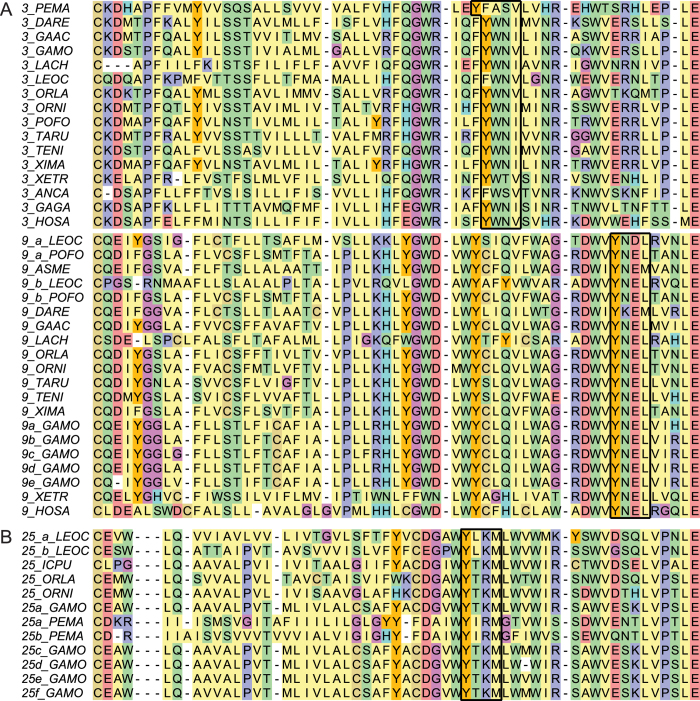
Edited amino acid alignments of the linker and transmembrane region of *TLR3*, *TLR9* and *TLR25* displaying known or putative tyrosine-containing endolysosomal sorting signals. (**A**) The known *TLR3* endolysosomal sorting signal is well conserved across species (black box) with the exception of *TLR3* in lamprey which has a phenylalanine in the tyrosine position and a tyrosine in the position before. For *TLR9* the signal is conserved in all species (black box). (**B**) For *TLR25* we propose an endolysosomal sorting signal in the linker region conserved across all species investigated that contain *TLR25*.

We compared known endolysosomal sorting signals from mammalian *TLRs* in the transmembrane, linker and cytosolic region against the corresponding regions of Atlantic cod *TLRs*. We found that the sorting signal in *TLR3* and *TLR9* were well conserved across all investigated species with the exception of *TLR3* in lamprey (Fig. 4A). We also searched for similar signals in the remaining *TLRs*: *TLR7*, *TLR8*, *TLR14*, *TLR21*, *TLR23* and *TLR25*. For *TLR25* a putative sorting signal was found (Fig. 4B), but for the other *TLRs* no clear conserved signalling motifs could be discerned (data not shown).

### Protein structure modelling and diversifying selection

**Figure 5:**
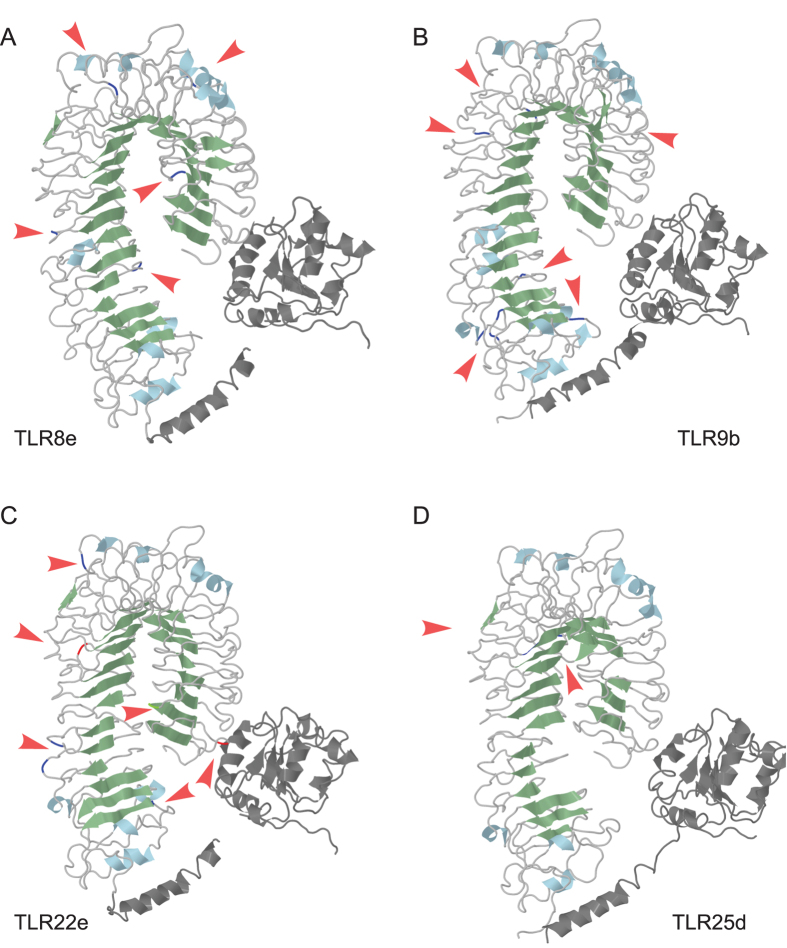
Sites under diversifying selection mapped onto the protein modeled structures of one paralog from each of the gene expansions *TLR8*, *TLR9*, *TLR22* and *TLR25* in Atlantic cod. The transmembrane, linker and TIR domain is colored dark grey whereas the ecto-domain is colored light grey with its sheets in pale green and helices in light blue. Sheets overlap with leucine-rich repeats in the ecto-domain. Arrows pointing at bright blue/bright red/bright green represents sites under diversifying selection as reported in Table 1. (**A**) Five sites (blue) mapped onto the modeled structure of *TLR8e*.The five sites are located both within and on the surface of the ecto-domain. (**B**) Eight sites (blue) mapped onto *TLR9b*. The sites are mainly located to two clusters in the ecto-domain with one cluster right at the border towards the transmembrane domain and one cluster in the middle of the ecto-domain. The sites are located both within and on the surface of the structure. (**C**) One, three and four sites (green, red and blue, respectively) are mapped onto *TLR22e*. With the exception of one site at the tip of the ecto-domain, the sites are located to the first half of the ecto-domain, mainly on the outer surface of the ecto-domain surface. (**D**) Two sites (blue) mapped onto *TLR25d* located to the middle and within the ecto-domain.

We modelled the 3D protein structure of all full-length *TLRs* in Atlantic cod (excluding those in Supplementary Table 4) onto the mammalian TLR5 structure (Fig. 5, Supplementary Figs 1, 2 and 3) as the overall structure of the TLR protein is central to TLR function. All modelled *TLRs* conformed to the overall TLR structure with a solenoid ecto-domain, transmembrane domain, linker and Toll/interleukin-1 receptor (TIR) domain. *TLR3*, *TLR7*, *TLR8*, *TLR9*, *TLR21*, *TLR22* and *TLR23* displayed a longer solenoid ecto-domain structure (Fig. 5, Supplementary Figs 1 and 2). *TLR14* and *TLR25* demonstrated a somewhat shorter structure with loops modelled in their ecto-domains - more similar to the structure of other plasma membrane TLRs in mammals (Supplementary Figs 2 and 3).

The expanded Atlantic cod *TLRs*, with the exception of *TLR7* due to low copy number, were analyzed for sites under selection using three phylogeny-guided methods; SLAC, FEL and REL (see methods for details and Table 1). *TLR22* appears to have the most sites under diversifying selection and *TLR25* the least. Sites common between two or more selection analyses were mapped onto one of the modelled protein structures for each of the *TLR8*, *TLR9*, *TLR22* and *TLR25* gene expansions demonstrating that the sites are mainly located to loops interspersed between the leucine-rich repeat elements in the *TLRs* ecto-domains (Fig. 5A–D).

### The TLR signalling pathway is intact in Atlantic cod

**Figure 6:**
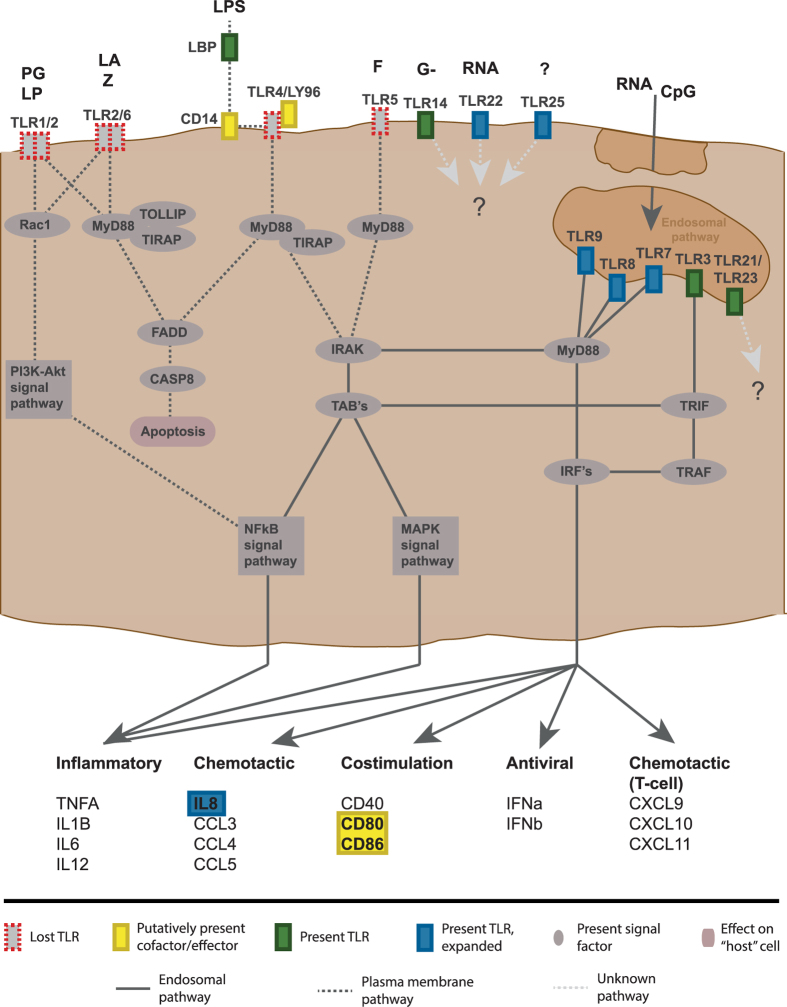
The mammalian TLR signaling pathway as depicted in KEGG condensed and presented to fit the proposed situation in Atlantic cod. Ligands are: PG – peptidoglycan (gram positive bacteria), LP – lipoprotein, LA – lipoarabinomannan, Z – zymosan (yeast), LPS – lipopolysaccharide (gram negative bacteria), G- – gram negative bacteria, F – flagellin, CpG – umethylated CpG DNA from bacteria. *TLR1/6*, *TLR2*, *TLR4* and *TLR5* are not found in Atlantic cod (also see Figs. 1 and 7). The presence of CD14, LY96 and CD80/86 was difficult to determine and are thus marked as putative. *TLR14*, *TLR21*, *TLR22*, *TLR23* and *TLR25* have unknown signaling pathways, but are drawn at their most likely affiliated membranes with the exception of *TLR22* drawn at the plasma membrane due to incongruent reports.

Using the mammalian TLR signalling network we searched for homologous genes in the new version of the Atlantic cod genome assembly (Supplementary Table 5). All components of the TLR signalling pathway were detected with the exception of TLR4 associated co-factors and some downstream T-cell/B-cell co-stimulatory molecules which were difficult to confirm due to distant sequence homology (Fig. 6). One downstream cytokine, interleukin-8 (*IL8*) showed substantial gene expansion: eight copies in total of which six were assembled to full-length (Supplementary Table 6). The translated sequences were subjected to a maximum likelihood (ML) protein sequence phylogenetic analysis together with *IL8* from fugu, tetraodon, tilapia, stickleback, medaka and human. The phylogeny grouped Atlantic cod *IL8’s* in two clades (Supplementary Fig. 4). Transcriptome profiling of *IL8* (identical to that performed on Atlantic cod *TLRs*) did not resolve the paralogs sufficiently and thus the expression pattern of each clade or individual paralogs could not be further addressed (data not shown).

### TLR annotation and vertebrate repertoires

**Figure 7:**
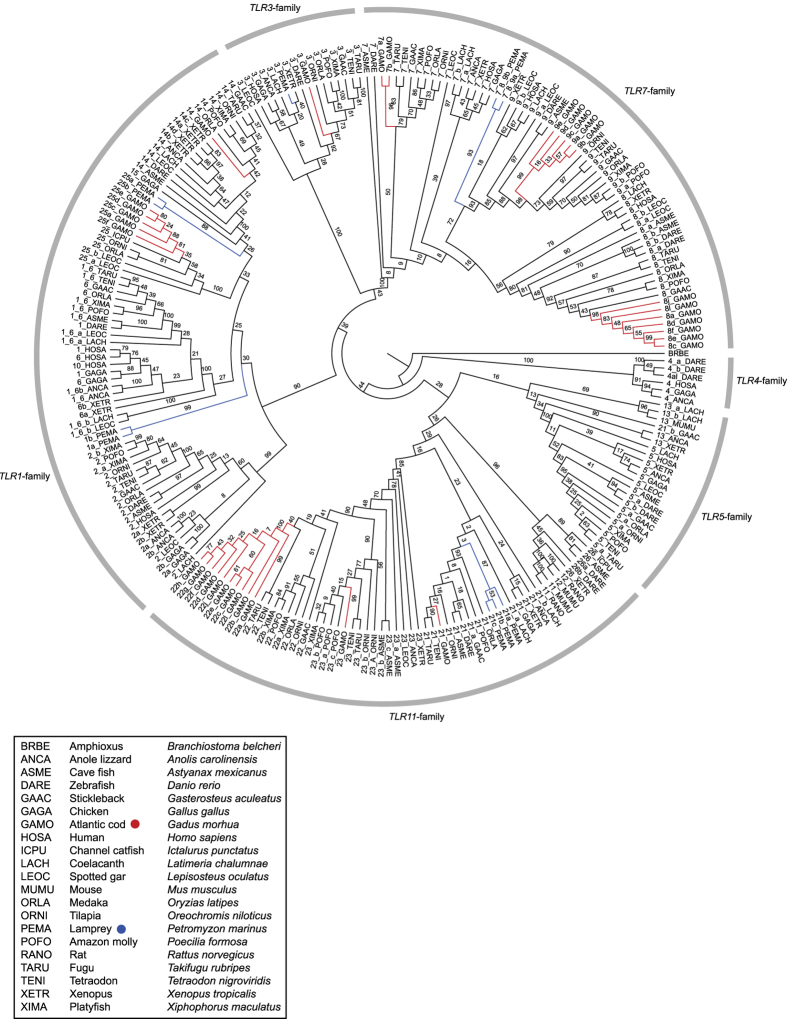
A ML-phylogeny made from the transmembrane, linker and TIR-domains from all full length *TLRs* found in all investigated vertebrate species listen in S1 Table 3 displayed with bootstrap values (see also Table 2). An Amphioxus *TLR* gene was used as the root. Atlantic cod genes are marked in red and lamprey in blue. The six major *TLR* families are marked with grey bars with corresponding family name. The Atlantic cod expansions are extreme compared to other teleost. Xenopus contains the largest expansion in addition to Atlantic cod with 4 copies of *TLR14*. Humans do not have representatives from the *TLR11*-family. Atlantic cod and lamprey do not have *TLR5*-family members. Atlantic cod is the only species without *TLR1/6* and *TLR2*. Some TLRs are only found in some species such as *TLR4*, *TLR10*, *TLR13*, *TLR15*, *TLR25* and *TLR26*. The resolution of the *TLR5*- and *TLR11*-families is somewhat poor compared to the other families due to the placement of *TLR13*, *TLR21* and *TLR26*.

We performed a multi-*TLR*, multi-species phylogenetic analysis using the translated sequence of the transmembrane, linker and TIR-domain regions of all *TLR* genes in selected vertebrate species with a main emphasis on teleosts (Supplementary Tables 2–4). The phylogeny resolved all six major *TLR* families, however, the *TLR11* and *TLR5* families display weaker support than the remaining families likely connected to the placement of *TLR21*, *TLR26* and *TLR13* (Fig. 7). Atlantic cod was the only species not harbouring any *TLRs* phylogenetically grouping within the *TLR1/6* and the *TLR2* clades of the *TLR1*-family. However, *TLR14* and *TLR25* are well supported within the *TLR1*-family clade. *TLR14* was not found in chicken and human. *TLR13* was present in the anole lizard (*Anolis carolinensis*), xenopus (*Xenopus tropicalis*) and coelacanth (*Latimeria chalumnae*). *TLR25* and *TLR26* were both sparsely found among the investigated fish species. Humans were the only species not displaying any members of the *TLR11*-family. The *TLR5*-family was not represented in either Atlantic cod or lamprey and the *TLR4*-family was only found in zebrafish, chicken (*Gallus gallus*), anole lizard and humans. Furthermore, the phylogeny demonstrates that the *TLR* gene expansions in Atlantic cod are rather extreme compared to the relatively few duplicates, triplicates and a single quadruplet expansion (xenopus *TLR14*) seen in the other species. No expansions were found within the human *TLR* repertoire (Fig. 7, Table 2).

## Discussion

### Signs of compensatory mechanisms for lost TLRs

Our *TLR* phylogeny indicates that Atlantic cod is the only known species lacking *TLR1/6* and *TLR2* which is confirmed by gene synteny analysis (Figs 1 and 7). These *TLRs*, members of the *TLR1*-family, are known to recognize peptidoglycan/lipoproteins at the plasma membrane. Roach *et al.*^8^ have demonstrated a convincing link between phylogenetic relationships and function within vertebrate TLR families. Our *TLR* phylogeny suggests that Atlantic cod has other representatives within the *TLR1*-family – *TLR14* and *TLR25* – and thus any reduced ability to detect peptidoglycan/lipoprotein by TLRs could be alleviated (Fig. 7). Our phylogeny and synteny analyses also describe the loss of *TLR5* in Atlantic cod, a plasma membrane associated TLR detecting flagellin^7^,^8^. However, no compensatory mechanism similar to that of the *TLR1*-family was found as no other Atlantic cod *TLR* was placed within the *TLR5*-family (Figs 1 and 7). However, due to overlapping ligand profiles flagellin detection is likely covered by other PRR families in this species - see^16^.

### Functional assessment of *TLRs* through comparative analyses

With the aim of inferring function on Atlantic cod *TLRs* we performed several comparative analyses based on sequence homology which we interpreted using established links between function and phylogenetic relationships, protein structure and sorting signals. For *TLR3*, *TLR7*, *TLR8* and *TLR9* our findings support earlier functional reports demonstrating nucleic acid ligands and intracellular localization identical to their mammalian counterparts (Figs 2,4A,5A,5B and 7 and Supplementary Fig. 2)^17^. There are limited functional studies on non-mammalian TLRs (TLR11–26) of which TLR14–26 are present in teleosts. For TLR14 and TLR25 functional studies have so far not fully resolved ligand specificity. However, interesting results include transcriptional up-regulation of *TLR14* after exposure to viable gram negative bacteria^18^ and transcriptional up-regulation of *TLR25* in response to parasites^19^. We propose a *TLR1*-family-like function for *TLR14* and *TLR25* implying plasma membrane localization and peptidoglycan or lipopolysaccharide-like ligands. This is further supported by protein structure modelling resolving shorter disrupted solenoid structures (Supplementary Figs 2 and 3) – structures correlated with plasma membrane localization and non-nucleic acid ligands^7^,^20^, Furthermore, the presence of an intact TLR signalling pathway (Fig. 6) also supports the proposed function of TLR14 and TLR25. Otherwise one would expect a concurrent loss of adaptor proteins and co-factors specific for plasma membrane associated TLR proteins – in line with the observed loss of all TLR4-associated adapters in species lacking *TLR4*^21^. Lastly, our analysis revealed a putative endolysosomal sorting signal in *TLR25* similar to that of mammalian TLR3 and TLR9 (Fig. 4B)^22^,^23^,^24^,^25^. For TLR21 reports suggest that it is an intracellular TLR with a nucleic acid ligand^26^,^27^. No firm conclusion can be drawn for TLR22; there are several incongruent reports indicating a cell surface location with a nucleic acid ligand as well as transcriptional response towards several non-nucleic acid stimulants like peptidoglycan and lipopolysaccharide^28^,^29^,^30^,^31^,^32^. The function of TLR23 is also not established^29^. *TLR21*, *TLR22* and *TLR23* all belong to the *TLR11*-family (Fig. 7) and display the longer solenoid structures indicative of intracellular localization and nucleic acid ligands (Supplementary Figs 1 and 2). Considering that the rodent-specific TLR11 and TLR12 of the *TLR11*-family is shown to have endosomal localization and that computational data supports a nucleic acid ligand for TLR22, our findings suggest that this whole family of TLRs do have nucleic acid ligands and most like intracellular localization^28^,^33^,^34^,^35^.

### Functional implications of lost and expanded *TLRs*

We detected diversifying selection among paralogs within the expanded Atlantic cod *TLRs*: *TLR8*, *TLR9*, *TLR22* and *TLR25* (Table 1). *TLR9* and *TLR22* stand out with the highest number of sites reported. Upon PAMP recognition, TLRs form TLR-homodimer:ligand complexes^36^. Vertebrates can further expand their detectable ligand repertoire by forming heterodimers within or between TLR families as have been demonstrated for TLR1/2, TLR2/6, TLR11/12 and TLR4/6^37^,^38^,^39^,^40^,^41^. The number of sites under diversifying selection in the ecto-domain of *TLR9* and *TLR22* suggests that the Atlantic cod’s innate immune strategy partly involves an increase in its detectable ligand repertoire relative to other investigated fish species through “heterodimerization” between paralogs or possibly heterodimerization of paralogs with other TLRs. For *TLR8* and *TLR25*, the number of sites detected was much lower and somewhat inconsistent between the different methods (Table 1) suggesting that increased detectable ligand repertoire is not the main force maintaining these two gene expansions. We investigated the possibility of increased gene dosage by performing a transcriptome profiling of all *TLRs* expressed in the spleen/head kidney of healthy juvenile Atlantic cod. Here we found no evident need of increased gene dosage, however, it suggests more tissue-specific *TLR* and *TLR* paralog usage (Fig. 3). This is supported by *TLR* expression analyses by Sundaram *et al.*^29^ in Atlantic cod (including *TLR22* paralogs) and by different expression levels of *TLRs* in various tissues in zebrafish and chicken^30^,^42^.

### Teleost *TLR* repertoires are more diverse compared to other vertebrates

Our phylogenetic analysis of vertebrate *TLRs* revealed substantial variation in *TLR* repertoires. All investigated fish species, except zebrafish, lack representatives of the *TLR4*-family, *TLR5* is not found in lamprey and Atlantic cod and *TLR22* is lost in zebrafish (Figs 2 and 7 and Table 2). In contrast, certain *TLRs* are only present in a few species independent of phylogenetic relationships – i.e. *TLR13, TLR23*, *TLR25* and *TLR26*. With regard to the gene expansions observed, duplications seems to be more frequent within teleosts and less frequently occurring in other vertebrate lineages (Fig. 7 and Table 2). This pattern may be connected to the teleost genome duplication event where a causal connection between gene/genome duplication and subsequent neofunctionalization of paralogs has been established in contrast to the usual reciprocal loss of gene duplicates^43^. This is also in line with the sites under diversifying selection detected in the Atlantic cod *TLR* expansions (Table 1). Our data also demonstrate that *TLR14* is lost from birds and humans and that humans lack the entire *TLR11*-family. Notably, the *TLR* diversity and phylogeny suggest that life history strategies involving aquatic life stages require a different array of *TLR11*-family members and additional *TLRs* from the *TLR1*-family (Fig. 7 and Table 2). Thus, the transition from an aquatic to a terrestrial lifestyle is associated with a shift in TLR repertoires – a shift that likely is linked to a highly different selection pressure on *TLRs* in the two environments.

### The birth-and-death of *TLRs*

Multigene families connected to the immune system tend to follow a birth-and-death (BD) evolutionary model promoting diversification that manifests as general phylogenetic interspecific gene clustering patterns, the presence of pseudogenes and gene losses^44^,^45^. Furthermore, gene expansions subjected to BD evolution and strong purifying selection undergo functional differentiation of the paralogs via sub- or neofunctionalization^44^. *TLRs* in general and especially their TIR-domains and leucine-rich repeat elements are known to be under strong purifying selection^46^,^47^,^48^. Our vertebrate *TLR* phylogeny demonstrates that gene losses and expansions are common in most lineages. However, the pattern is less pronounced in non-teleost lineages. Among teleosts, Atlantic cod shows the most pronounced loss and expansion pattern (Fig. 7 and Table 2). The BD model further supports our finding that sites under diversifying selection within *TLR8* and *TLR22* (and possibly *TLR9* and *TLR25*) in Atlantic cod (Table 1) likely increase the detectable ligand repertoire in this species. Finally, the extreme case of Atlantic cod compared to other teleosts indicates that its *TLR* repertoire is associated with the loss of *MHC-II*, i.e. that the loss of such a major adaptive immune system component has boosted evolutionary innovation through interlinked gene losses and expansions leading to high complexity and greater relative dependence on the innate immune system in this species.

## Materials and Methods

### Genome assembly

The genome assembly used in this study is one of four assemblies used to produce a new release of the Atlantic cod genome (Tørresen & Nederbragt *et al.* in prep). In short, overlapping sequencing reads from Illumina (180 bp insert size, 100 nt read length) were merged with FLASH using default options^49^. Meryl and merTrim were used to count and correct the reads, both programs from the Celera Assembler package 8.1^50^. 454 reads used in Star *et al.*^13^ were converted from .sff files with sffToCA (also from Celera Assembler package) and corrected with merTrim, before trimmed with overlap based trimming (OBT, Celera Assembler program). Celera Assembler 8.2 alpha was used to trim subreads of PacBio sequencing reads. 20x of the merged Illumina 180 bp insert size reads, all paired 454 reads and the trimmed PacBio reads were used in an assembly with the Celera Assembler. The resulting genome assembly had some gaps closed with PBJelly^51^ and was polished by Pilon^52^. Details are available upon request and later in Tørresen & Nederbragt *et al.* (in prep).

### Genome mining for PRRs

We searched for PRR genes representing the major PRR families known in mammals listed in Supplementary Table 1 collected from Ensembl and UniProt^53^,^54^. The search was performed using TBLASTN from the BLAST+ suite with an e-value cut-off of 1e−1^55^. The low e-value was used to capture distant sequence homologs. Homologous relationships are described in Supplementary Table 1.

### Selection of full-length TLR genes for further analyses

Annotated *TLR* sequences from selected species in Ensembl and GenBank covering all known *TLR* genes (listed in Supplementary Table 2) were compared towards the Atlantic cod genome using TBLASTN from the BLAST+ suite with an e-value cut-off of 1e−10 and otherwise default parameters^53^,^55^,^56^. All putative contigs containing *TLRs* were loaded into MEGA5^57^ where regions of interest in each scaffold were extracted. Only full-length *TLRs* containing a complete ecto-domain, transmembrane domain, linker and complete TIR-domain were evaluated further. We performed RNAseq to evaluate expression levels as some of the full-length *TLR*s extracted contained several insertions and deletions making poor translated protein sequences. All extracted full-length *TLRs* were used to make an Atlantic cod *TLR* index. The quality and adapter trimmed RNAseq sequences from six healthy juvenile Atlantic cod (see RNAseq method section) were mapped towards this database and raw counts extracted using the RSEM/Bowtie wrapper included in Trinity v2.0.6^58^. These raw counts were normalized using the included edgeR scripts in Trinity to obtain TMM normalized FPKM counts^59^. *TLRs* with large amounts of insertions/deletions, either alone or in combination with low read counts, were excluded from further analysis as the accuracy of the translate protein sequences was questionable (Supplementary Table 4). Count matrix is available in the GitHub repository (https://github.com/uio-cels/Solbakken_TLRs).

### Fish and totalRNA isolation for RNA sequencing

Total RNA was isolated from the head kidney/spleen of six healthy juvenile Atlantic cod. These fish originate from the Norwegian cod breeding program and were reported to be healthy without any history of diseases. The use of live Atlantic cod was approved by the National Animal Research authority in Norway (FOTS id 1147) and all methods were in accordance with the approved guidelines. The fish were transported at approx. 2 g to 100 L tanks at the Aquaculture Research Station (Tromsø, Norway) for grow-out in seawater of 3.4% salinity at 10 °C, 24 hour light and fed *ad libitum* with commercial feed (BioMar, Norway). The rates of water inflow were adjusted to an oxygen saturation of 90–100% in the outlet water. The tissue was stored on RNAlater (Life Technologies) and total RNA was isolated using Trizol (Life Technologies) according to protocol but using half the amount of tissue per volume Trizol recommended by the manufacturer. The complete laboratory protocol is available in the GitHub repository (https://github.com/uio-cels/Solbakken_TLRs). Sequencing libraries were produced according to the IlluminaTruSeq protocol (Illumina, Inc., San Diego, CA). Illumina HiSeq2000 100 bp paired-end sequencing services were provided by the Norwegian Sequencing Centre (http://www. sequencing.uio.no). Sequences were trimmed for adapters using Cutadapt v1.0 and trimmed on quality using Sickle using known Illumina adapter sequences, a Q threshold of 20 and otherwise default parameters^60^,^61^.

### Synteny analyses

The Ensembl^53^ genome browser v78 (unless otherwise stated) was used to chart annotated open reading frames around *TLRs* annotated in the selected fish species. Protein sequences from these genes were downloaded and used in a TBLASTN^55^ towards the Atlantic cod genome together with *TLR* representatives with an e-value cut-off of 1e−10. If a certain *TLR* was not annotated in one or several of the selected fish genomes in Ensembl we used the Ensembl BLAST tool with protein queries towards nucleic acid resources (TBLASTN) with default parameters to find the genomic region of interest. Some genome regions were reverse complemented for figure. drawing purposes and this is noted in the respective figures (Figs 1 and 2).

### Endolysosomal sorting signals

Characterized *TLR* sorting signals were obtained from the literature^22^,^23^. Protein sequence was obtained for all *TLR3* and all *TLR9* genes investigated in this study (Supplementary Table 2). These were aligned with default settings using MEGA5 and ClustalW (Fig. 4A)^57^. We also searched for similar tyrosine based signals in the linker region of the remaining Atlantic cod *TLRs* (*TLR7*, *TLR8*, *TLR14*, *TLR21*, *TLR22*, *TLR23* and *TLR25*) (Fig. 4B).

### TLR signalling pathway

The mammalian TLR signalling pathway available through the KEGG database^62^ was used as a basis for mapping the pathway components in the Atlantic cod genome. The connected UniProt sequences for each pathway component were used in a TBLASTN search together with annotated homologs from fish species available at Ensembl or UniProt (Supplementary Table 5) towards the Atlantic cod genome with an e-value cut-off of 1e−1^53^,^54^,^55^. The low e-value was used due to distant homology of sequences between fish and mammals. Genes that were difficult to verify are highlighted in Fig. 6.

### Protein structure prediction

Translated Atlantic cod *TLR* sequences were submitted to the Phyre2 structure prediction server for modelling^63^. All sequences were modelled against TLR5. All TLRs from *Homo sapiens* (human), *Petromyzon marinus* (lamprey), *Anolis carolinensis* (lizard) and *Oreochromis niloticus* (tilapia) were also submitted to Phyre2 and modelled onto the human TLR5 crystal structure (Fold library id: c3j0aA). The structures were coloured for visualization purposes using Jmol^64^, differentiating between loops, sheets and helices as well as the transmembrane, linker and TIR-domain (Supplementary Table 2 and Supplementary Figs 1–3). All Atlantic cod PDB files are available in the GitHub repository (https://github.com/uio-cels/Solbakken_TLRs).

### Selection analyses

The expanded Atlantic cod *TLR*s with three or more full-length copies (*TLR8*, *TLR9*, *TLR22* and *TLR25*) were analyzed using Datamonkey^65^. Nucleotide sequences were imported into MEGA5 for alignment using default ClustalW parameters. The alignment was then manually edited to ensure proper translation to amino acids. A maximum likelihood phylogeny was made using partial deletion, a Jukes-Cantor model of sequence evolution with gamma distributed rate heterogeneity^57^. The resulting phylogeny was submitted together with the nucleotide alignment to Datamonkey. For each *TLR* expansion a model test was first run. The proposed best model was used before running selection analyses with the SLAC, FEL and REL methods. These are codon based maximum likelihood methods estimating rates of nonsynonymous and synonymous changes at each site in an alignment to identify sites under positive or negative selection. These tests are originally designed to be run on interspecies alignments. Here, since the tests are run on intraspecies paralogs, we argue that the sites reported to be under positive selection actually are under diversifying selection. The term diversifying selection is thus used throughout this report. Fixed effects likelihood model (FEL) estimates the ratio of nonsynonymous to synonymous substitution rates for each site in a sequence alignment with fixed estimates for branch lengths and substitution rate bias parameters. Random effects likelihood model (REL) allows rate variation in both nonsynonymous and synonymous rates and a general underlying nucleotide substitution model. Single-likelihood ancestor counting (SLAC) model weights the nucleotide substitution biases which are estimated from the data and allow ambiguous codons in the data. Sites reported to be under diversifying selection in two or more tests are highlighted in one of the protein structure models made for each of the *TLR8*, *TLR9*, *TLR22* and *TLR25* expansions. In cases where only one test has reported sites it is noted in the Fig. legend (Table 1 and Fig. 5). Phylogenies and alignments are available in the GitHub repository (https://github.com/uio-cels/Solbakken_TLRs).

### Vertebrate *TLR* phylogeny

Full-length protein sequences were not alignable due to large variations in the ecto-domain of the *TLR*s. Thus, the transmembrane region, linker and TIR-domain were used as basis for phylogenetic analysis after alignment and minor curation of the data using MEGA5^57^. PROTTEST^66^ was used for substitution model optimalization with the Bayesian Information Criterion (BIC) model selection criterion and testing all seven models available. PROTTEST suggested the JTT+I+G+F as the best substitution model. A maximum likelihood tree was produced using Randomized Axelerated Maximum Likelihood (RAxML) HPC-PTHREADS version 7. 2. 6 with the PROTCATJTT model^67^. The rapid bootstrap/search for the best tree simultaneously option was used and the analysis was run with 500 bootstraps. The resulting phylogeny was used as the basis for the final *TLR* annotations of all sequences used and described in this study (Supplementary Table 2). The tree was imported into FigTree v1.4^68^ for cladogram transformation and then edited in Adobe Illustrator for improved Fig. visualization (Fig. 7). The alignment is available in the GitHub repository (https://github.com/uio-cels/Solbakken_TLRs).

## Additional Information

**How to cite this article**: Solbakken, M. H. *et al.* Evolutionary redesign of the Atlantic cod (*Gadus morhua* L.) Toll-like receptor repertoire by gene losses and expansions. *Sci. Rep.* **6**, 25211; doi: 10.1038/srep25211 (2016).

## Supplementary Material

Supplementary Information

## Figures and Tables

**Table 1:** Sites under diversifying selection as reported by SLAC, FEL and REL analyses.

Analysis	TLR8	TLR9	TLR22	TLR25
SLAC	0	0	3	0
FEL	5	9	27	2
REL	0	44	7	0
Common sites	0	8*	1/3/4**	0

^*^Sites reported that are common between FEL and REL.

^**^Sites reported that are common between all, SLAC and FEL or FEL and REL respectively.

**Table 2:** Overview of the full length *TLRs* found in all investigated species.

	TLR1	TLR2	TLR6	TLR10	TLR14	TLR15	TLR25	TLR3	TLR4	TLR5	TLR13	TLR7	TLR8	TLR9	TLR21	TLR22	TLR23	TLR26
Homo sapiens	x	x	x	x				x	x	x		x	x	x				
Gallus gallus	x	x^2	x			x		x	x	x		x			x			
Anolis carolinensis	x^2	x^2	?		x	?		x	x	x	x	x			x		x	
Xenopus tropicalis	x^2	x^2	x		x^4			x		x	x	x	x	x	x		x	x
Gadus morhua					x		x^5(7)	x				x^2(3)	x^7(12)	x^5	x	x^8(12)	x	
Oreochromis niloticus	Frag.	x	?		x		x	x		x^2		x	x	x	x	x	x^2	
Poecilia formosa	x	x	?		x			x		x		x	x	x^2	x	x	x^3	
Takifugu rubripes	x	x	?		x			x		x^2		x	x	x	x	x	x	
Tetraodon nigroviridis	x	x	?		x			x		x		x	x	x	x	x	x	
Xiphophorus maculatus	x	x^2	?		x			x		x		x	x	x	x	x^2	x	
Astyanax mexicanus	x	x			x			x		x		x	x^2	x	x		x^3	x
Lepisosteus oculatus	x^2	x	?		x		x^2	x		x		x	x	x^2			x	
Gasterosteus aculeatus	x	x	?		x			x		x^3		x	x	x	x^2	x		
Oryzias latipes	x	x	?		x		x	x		x^2		x	x	x	x	x		
Danio rerio	x	x			x			x	x^3^1^	x^2		x	x^2	x	x			x^2
Latimeria chalumnae	x^2	x	?		x			x		x	x^2	x^2	x	x	x^2			
Petromyzon marinus	x^2		?		x		x^2	x				x^2			x^3			

Caret (^): the number of copies for a given gene if expanded. For *Gadus morhua* the number presented within () includes the genes excluded from further analyses given in S1 Table 4. For *TLR1* and *TLR6* – if homology could not be determined with confidence the copy was assigned to *TLR1* and a? designation given for *TLR6*. 1TLR4 in zebrafish does not have homologous function to mammalian TLR4 (see reference Sepulcre, *et al.* 2009).
