# Supplementary material for: Evolutionary redesign of the Atlantic cod (Gadus morhua L.) Toll-like receptor repertoire by gene losses and expansions
Source: Sci Rep. 2016 Apr 29;6:25211. doi: 10.1038/srep25211 (PMC4850435; doi:10.1038/srep25211)
Supplement: Supplementary Information [file srep25211-s1.pdf]

Supplementary information

Evolutionary redesign of the Atlantic cod (*Gadus morhua* L.) Toll-like receptor repertoire by gene losses and expansions

Monica H. Solbakken[1*], Ole K. Tørresen[1], Alexander J. Nederbragt[1], Marit Seppola[2,], Tone F. Gregers[3], Kjetill S. Jakobsen[1], Sissel Jentoft[1,4]

Supplementary table 1 – An overview over all genes searched for belonging to the most common PRR families. Putative hits indicate that phylogenetic analyis should be performed to determine family member.

Gene	Query species	Pathway	Blast hit comment	Blast hit region Celera140828_ pbjellypilon
AIM2	HOSA, MUMU	NOD-like receptor, Pattern recognition receptor (PRR)	No clear sequence homology	
APCS	HOSA, DARE, MUMU, XETR	Pentraxins - Pattern recognition receptor (PRR)	No clear sequence homology	Does overlap somewhat with CRP hits
CD14	HOSA, MUMU, XETR	Pattern recognition receptor (PRR)	No clear sequence homology	
CLC4E	HOSA, MUMU, XETR	C-type lectin domain family	No clear sequence homology	
CLC6A	HOSA, MUMU, TARU, TENI, GAAC	C-type lectin domain family	No clear sequence homology	
CLC7A	HOSA, MUMU	C-type lectin domain family	No clear sequence homology	
COLEC10	HOSA, GAAC, TARU	Collectin - C-type lectin Pattern recognition receptor (PRR)	Yes	Contig1376_pilon
COLEC11	GAMO, HOSA, GAAC, TARU	Collectin - C-type lectin Pattern recognition receptor (PRR)	yes	Contig847_pilon
COLEC12	GAMO, GAAC, TARU, HOSA	Collectin - C-type lectin Pattern recognition receptor (PRR)	yes	Contig2237_pilon, Contig2157_pilon, Contig298_pilon
CRP	GAMO, DARE, HOSA, MUMU, SASA, XETR	Pentraxins - Pattern recognition receptor (PRR)	Yes, but weak homology	Contig1070_pilon, Contig1499_pilon, Contig1934_pilon, Contig2939_pilon, Contig5417_pilon, Contig805_pilon, Contig7743_pilon, Contig1220_pilon

Ficolin1, 2, 3, A/B	HOSA, MUMU	Patter recognition receptor, collectin family, complement activating	Yes, putative	Contig1126_pilon, Contig9321_pilon
FP	CRIMI	Pentraxins - Pattern recognition receptor (PRR)	No clear sequence homology	Does overlap somewhat with CRP hits
LGP2	GAMO, GAAC, DARE, HOSA, TARU	RIG-like receptors Pattern recognition receptor (PRR)	Yes	Contig795_pilon
MBL2	HOSA, MUMU, ONMY, SASA	Pattern recognition receptor, Collectin, mannose binding lectin complement activating	No clear sequence homology	
MDA5	DARE, GAAC, HOSA, TARU	RIG-like receptors Pattern recognition receptor (PRR)	Yes	Contig5186_pilon, Contig795_pilon, Contig2519_pilon
MRC1	HOSA, MUMU, XETR	Macrophage mannose receptors Pattern recognition receptor (PRR)	Yes	Contig2237_pilon
MRC2	GAAC, GAMO, HOSA, MUMU, TARU, XETR	Macrophage mannose receptors Pattern recognition receptor (PRR)	Yes	Contig6711_pilon
MSR1	HOSA, MUMU, XETR	Macrophage scavenger receptors Pattern recognition receptor (PRR)	Yes, expanded	Contig8844_pilon, Contig8342_pilon, Contig309_pilon, Contig2235_pilon, Contig830_pilon, Contig208_pilon, Contig669_pilon
NAIP	HOSA, MUMU, XETR	NOD-like receptor, Pattern recognition receptor (PRR)	Yes, but weak homology	Contig50_pilon
NLRC3	GAMO, HOSA, TARU	NOD-like receptor CARD domain containing, Pattern recognition receptor (PRR)	Yes	Contig1688_pilon
NLRC4	HOSA, XETR	NOD-like receptor CARD domain containing, Pattern recognition receptor (PRR)	No clear sequence homology	
NLRC5	HOSA, GAAC, XETR	NOD-like receptor CARD domain containing, Pattern recognition receptor (PRR)	No clear sequence homology	
NLRP1	HOSA, MUMU	Pattern recognition receptor, NOD like pyrin containing family	No clear sequence homology	
NLRP10	HOSA, MUMU	Pattern recognition receptor, NOD like pyrin containing family	No clear sequence homology	
NLRP11	HOSA	Pattern recognition receptor, NOD like pyrin containing family	No clear sequence homology	
NLRP12	HOSA, MUMU	Pattern recognition receptor, NOD like pyrin containing family	No clear sequence homology	
NLRP13	HOSA	Pattern recognition receptor, NOD like pyrin containing family	No clear sequence homology	

NLRP14	HOSA, MUMU	Pattern recognition receptor, NOD like pyrin containing family	No clear sequence homology	
NLRP2	HOSA	Pattern recognition receptor, NOD like pyrin containing family	No clear sequence homology	
NLRP3	HOSA, MUMU	Pattern recognition receptor, NOD like pyrin containing family	No clear sequence homology	
NLRP4	HOSA, MUMU	Pattern recognition receptor, NOD like pyrin containing family	No clear sequence homology	
NLRP5	HOSA, MUMU	Pattern recognition receptor, NOD like pyrin containing family	No clear sequence homology	
NLRP6	HOSA, MUMU	Pattern recognition receptor, NOD like pyrin containing family	No clear sequence homology	
NLRP7	HOSA	Pattern recognition receptor, NOD like pyrin containing family	No clear sequence homology	
NLRP8	HOSA	Pattern recognition receptor, NOD like pyrin containing family	No clear sequence homology	
NLRP9	HOSA, MUMU	Pattern recognition receptor, NOD like pyrin containing family	No clear sequence homology	
NLRX1	GAMO, GAAC, HOSA	NOD-like receptor, Pattern recognition receptor (PRR)	Yes	Contig450_pilon
NOD1	GAMO, GAAC, HOSA, ORLA, TARU	Nucleotide oligomerization domain (NOD) containing receptors Pattern recognition receptor (PRR)	Yes	Contig619_pilon
NOD2	HOSA, GAAC, ORLA	Nucleotide oligomerization domain (NOD) containing receptors Pattern recognition receptor (PRR)	No clear sequence homology	
PGRP_La1, La2, La3	TENI	Peptidoglycan_recognition_protein	Yes, unable to separate La1, LA2 and La3	Contig364_pilon, Contig1756_pilon
PGRP1	HOSA, MUMU	Peptidoglycan_recognition_protein	Weak sequence homology	Contig364_pilon, Contig1756_pilon
PGRP2	HOSA, MUMU	Peptidoglycan_recognition_protein	Weak sequence homology	Contig364_pilon, Contig1756_pilon
PGRP3	HOSA, MUMU	Peptidoglycan_recognition_protein	Weak sequence homology	Contig364_pilon, Contig1756_pilon
PGRP4	HOSA, MUMU	Peptidoglycan_recognition_protein	Weak sequence homology	Contig364_pilon, Contig1756_pilon
PGRP5	CTEID	Peptidoglycan_recognition_protein	Weak sequence homology	Contig364_pilon, Contig1756_pilon
PTX3	GAMO, HOSA, ORLA, TARU	Pentraxins - Pattern recognition receptor (PRR)	Yes	Contig249_pilon

RIG_I	HOSA, DARE, MUMU, SASA, XETR, CYPCA	RIG-like receptors Pattern recognition receptor (PRR)	No clear sequence homology	Hits LGP2 region
Surfactant protein A1/A2/D	HOSA	Collectin - C-type lectin Pattern recognition receptor (PRR)	No clear sequence homology	

Supplementary table 2 – Overview over all *TLR* sequences included in this study. *TLR1_GAGA was recently discontinued from the Ensembl database.

Gene_species	Identifiers	Source
Outgroup		
BRBE	Q1KVP8	Uniprot
TLR1/6/10		
TLR1_6a_LACH	ENSLACT00000011495	Ensembl v78
TLR1_6a_LEOC	ENSLOCT00000015924	Ensembl v78
TLR1_6_ASME	ENSAMXT00000026289	Ensembl v78
TLR1_6b_LACH	ENSLACT00000017309	Ensembl v78
TLR1_6b_LEOC	ENSLOCT00000021874	Ensembl v78
TLR1_6_POFO	ENSPFOT00000020197	Ensembl v78
TLR1_6_TARU	ENSTRUT00000025237	Ensembl v67
TLR1_6_TENI	ENSTNIT00000017441	Ensembl v67
TLR1_6a_ANCA	ENSACAT00000007334 + 2000bp 5'	Ensembl v67
TLR1_6b_ANCA	ENSACAT00000024231	Ensembl v67
TLR1_DARE	ENSDART00000063176	Ensembl v67
TLR1_GAGA*	ENSGALT00000037374	Ensembl v67
TLR1_HOSA	ENST00000502213	Ensembl v67
TLR1a_PEMA	ENSPMAT00000011445	Ensembl v67
TLR1b_PEMA	ENSPMAT00000011446	Ensembl v67
TLR6_GAAC	ENSGACT00000023764	Ensembl v67
TLR6_GAGA	ENSGALT00000028179	Ensembl v67
TLR6_HOSA	ENST00000381950	Ensembl v67
TLR6_ORLA	ENSORLT00000005546	Ensembl v67
TLR6a_XETR	ENSXETT00000057556	Ensembl v67
TLR6b_XETR	ENSXETT00000057561	Ensembl v67
TLR1_6_XIMA	JH556664.1:787041:791499	Ensembl v78
TLR10_HOSA	ENST00000308973	Ensembl v67
TLR2		
TLR2a_XIMA	ENSXMAT00000008287	Ensembl v78
TLR2_ASME	ENSAMXT00000026308	Ensembl v78
TLR2b_XIMA	ENSXMAT00000008292	Ensembl v78
TLR2_DARE	ENSDART00000122568	Ensembl v67
TLR2_GAAC	ENSGACT00000024730	Ensembl v67
TLR2_HOSA	ENST00000260010	Ensembl v67
TLR2_LACH	ENSLACT00000014405	Ensembl v78
TLR2_LEOC	ENSLOCT00000022362	Ensembl v78
TLR2_ORLA	ENSORLT00000003160	Ensembl v67
TLR2_ORNI	ENSONIT00000017759	Ensembl v67
TLR2_TARU	ENSTRUT00000007500	Ensembl v67
TLR2_TENI	Un_random:56715842-56719817	Ensembl v78
TLR2a_ANCA	ENSACAT00000027553	Ensembl v67
TLR2a_GAGA	ENSGALT00000015034	Ensembl v67
TLR2a_XETR	ENSXETT00000003822	Ensembl v67

TLR2b_ANCA	ENSACAT00000021129	Ensembl v67
TLR2b_GAGA	ENSGALT00000015036	Ensembl v67
TLR2b_XETR	ENSXETT00000003835	Ensembl v67
TLR2_POFO	ENSPFOT00000000534	Ensembl v78
TLR3		
TLR3_ANCA	ENSACAT00000001699	Ensembl v67
TLR3_DARE	ENSDART00000013021	Ensembl v67
TLR3_GAAC	ENSGACT00000022330	Ensembl v67
TLR3_GAGA	ENSGALT00000021952	Ensembl v67
TLR3_GAMO	Contig1920	Celera140828_pbjelly_pilon
TLR3_HOSA	ENST00000296795	Ensembl v67
TLR3_LEOC	ENSLOCT00000017076	Ensembl v78
TLR3_ORLA	ENSORLT00000010278	Ensembl v67
TLR3_ORNI	ENSONIT00000012803	Ensembl v67
TLR3_PEMA	ENSPMAT00000001785	Ensembl v67
TLR3_POFO	ENSPFOT00000005820	Ensembl v78
TLR3_TARU	ENSTRUT00000012255	Ensembl v67
TLR3_TENI	ENSTNIT00000010103	Ensembl v67
TLR3_XETR	ENSXETT00000012947	Ensembl v67
TLR3_XIMA	ENSXMAT00000018937	Ensembl v78
TLR3_LACH	ENSLACT00000013051	Ensembl v78
TLR3_ASME	ENSAMXT00000020816 partial	Ensembl v78
TLR4		
TLR4a_DARE	ENSDART00000124036	Ensembl v67
TLR4_ANCA	ENSACAT00000013935	Ensembl v67
TLR4b_DARE	ENSDART00000034852	Ensembl v67
TLR4_GAGA	ENSGALT00000011333	Ensembl v67
TLR4_HOSA	ENST00000355622	Ensembl v67
TLR4al_DARE	ENSDART00000113952	Ensembl v67
TLR5		
TLR5a_DARE	ENSDART00000148798	Ensembl v67
TLR5a_GAAC	ENSGACT00000005799	Ensembl v67
TLR5a_ORLA	ENSORLT00000020304	Ensembl v67
TLR5a_ORNI	ENSONIT00000001682	Ensembl v67
TLR5a_TARU	ENSTRUT00000011390	Ensembl v67
TLR5_ANCA	ENSACAT00000002427	Ensembl v67
TLR5_ASME	ENSAMXT00000026738	Ensembl v78
TLR5b_DARE	ENSDART00000150030	Ensembl v67
TLR5b_GAAC	ENSGACT00000009486 partial	Ensembl v67
TLR5c_GAAC	ENSGACT00000009484 partial	Ensembl v67
TLR5_GAGA	ENSGALT00000015302	Ensembl v67
TLR5_HOSA	ENST00000366881	Ensembl v67
TLR5_LACH	ENSLACT00000017589	Ensembl v78
TLR5_LEOC	ENSLOCT00000022142	Ensembl v78
TLR5_POFO	ENSPFOT00000000699	Ensembl v78
TLR5_TENI	ENSTNIT00000008908	Ensembl v67

TLR5_XETR	ENSXETT00000032872	Ensembl v67
TLR5_XIMA	ENSXMAT00000001788	Ensembl v78
TLR7/8/9		
TLR7_8_9a_PEMA	ENSPMAT00000002559	Ensembl v67
TLR7_8_9b_PEMA	ENSPMAT00000006917	Ensembl v67
TLR7a_GAMO	Contig1607	Celera140828_pbjelly_pilon
TLR7a_LACH	ENSLACT00000001169	Ensembl v78
TLR7_ANCA	ENSACAT00000001581	Ensembl v67
TLR7_ASME	ENSAMXT00000026786	Ensembl v78
TLR7b_GAMO	Contig1607	Celera140828_pbjelly_pilon
TLR7b_LACH	ENSLACT00000004799	Ensembl v78
TLR7c_GAMO	Contig8584	Celera140828_pbjelly_pilon
TLR7_DARE	ENSDART00000130393	Ensembl v67
TLR7_GAAC	ENSGACT00000005274	Ensembl v67
TLR7_GAGA	ENSGALT00000026777	Ensembl v67
TLR7_HOSA	ENST00000380659	Ensembl v67
TLR7_LEOC	ENSLOCT00000012218	Ensembl v78
TLR7_ORLA	21:26169462:26173343	Ensembl v67
TLR7_ORNI	GL831311.1:1118220:1122015	Ensembl v67
TLR7_POFO	ENSPFOT00000025479	Ensembl v78
TLR7_TARU	scaffold_103:438696-441905	Ensembl v67
TLR7_TENI	2:12682773-12685934	Ensembl v78
TLR7_XETR	ENSXETT00000011725	Ensembl v67
TLR7_XIMA	ENSXMAT00000004441	Ensembl v78
TLR8a_ASME	ENSAMXT00000026340	Ensembl v78
TLR8a_DARE	ENSDART00000124995	Ensembl v67
TLR8a_GAMO	Contig1607	Celera140828_pbjelly_pilon
TLR8a_LEOC	ENSLOCT00000012227	Ensembl v78
TLR8b_ASME	ENSAMXT00000001765	Ensembl v78
TLR8b_DARE	ENSDART00000110194	Ensembl v67
TLR8b_GAMO	Contig1607	Celera140828_pbjelly_pilon
TLR8b_LEOC	ENSLOCT00000012238	Ensembl v78
TLR8c_GAMO	Contig1607	Celera140828_pbjelly_pilon
TLR8d_GAMO	Contig1607	Celera140828_pbjelly_pilon
TLR8e_GAMO	Contig1607	Celera140828_pbjelly_pilon
TLR8f_GAMO	Contig1607	Celera140828_pbjelly_pilon
TLR8g_GAMO	Contig1607	Celera140828_pbjelly_pilon
TLR8h_GAMO	Contig1607	Celera140828_pbjelly_pilon
TLR8i_GAMO	Contig1607	Celera140828_pbjelly_pilon
TLR8j_GAMO	Contig1329	Celera140828_pbjelly_pilon
TLR8k_GAMO	Contig1329	Celera140828_pbjelly_pilon
TLR8l_GAMO	Contig8584	Celera140828_pbjelly_pilon
TLR8_GAAC	ENSGACT00000005251	Ensembl v67
TLR8_HOSA	ENST00000218032	Ensembl v67
TLR8_LACH	ENSLACT00000019040	Ensembl v78
TLR8_ORLA	21:26174329:26178070	Ensembl v67

TLR8_ORNI	GL831311.1:1102783-1105960 partial TIR in gap	Ensembl v78
TLR8_POFO	ENSPFOT00000023362	Ensembl v78
TLR8_TARU	scaffold_103:442965-446072	Ensembl v78
TLR8_TENI	2:12678635-12681709	Ensembl v78
TLR8_XETR	ENSXETT00000063320	Ensembl v67
TLR8_XIMA	ENSXMAT00000004440	Ensembl v78
TLR9a_GAMO	Contig57	Celera140828_pbjelly_pilon
TLR9a_LEOC	ENSLOCT00000017524	Ensembl v78
TLR9a_POFO	ENSPFOT00000000378	Ensembl v78
TLR9_ASME	ENSAMXT00000027071	Ensembl v78
TLR9b_GAMO	Contig57	Celera140828_pbjelly_pilon
TLR9b_LEOC	ENSLOCT00000017790	Ensembl v78
TLR9b_POFO	ENSPFOT00000023878	Ensembl v78
TLR9c_GAMO	Contig57	Celera140828_pbjelly_pilon
TLR9d_GAMO	Contig57	Celera140828_pbjelly_pilon
TLR9_DARE	ENSDART00000124218	Ensembl v67
TLR9e_GAMO	Contig57	Celera140828_pbjelly_pilon
TLR9_GAAC	ENSGACT00000013450	Ensembl v67
TLR9_HOSA	ENST00000360658	Ensembl v67
TLR9_LACH	ENSLACT00000021105	Ensembl v78
TLR9_ORLA	ENSORLT00000011109	Ensembl v67
TLR9_ORNI	ENSONIT00000015652	Ensembl v67
TLR9_TARU	ENSTRUT00000019252	Ensembl v67
TLR9_TENI	ENSTNIT00000018282	Ensembl v67
TLR9_XETR	ENSXETT00000013094	Ensembl v67
TLR9_XIMA	ENSXMAT00000007659	Ensembl v78
TLR11/12/13		
TLR11_MUMU	ENSMUST00000063570	Ensembl v67
TLR11_RANO	ENSRNOT00000039925	Ensembl v67
TLR12_MUMU	ENSMUST00000074829	Ensembl v67
TLR12_RANO	ENSRNOT00000065640	Ensembl v67
TLR13a_LACH	ENSLACT00000007245	Ensembl v78
TLR13_ANCA	ENSACAT00000016690	Ensembl v67
TLR13b_LACH	ENSLACT00000014715	Ensembl v78
TLR13_MUMU	ENSMUST00000040065	Ensembl v67
TLR13_XETR	ENSXETT00000009956	Ensembl v67
TLR14		
TLR14_ANCA	ENSACAT00000022679	Ensembl v67
TLR14_ASME	ENSAMXT00000019326	Ensembl v78
TLR14_DARE	ENSDART00000149289	Ensembl v67
TLR14_GAAC	ENSGACT00000002286	Ensembl v67
TLR14_GAMO	Contig2455	Celera140828_pbjelly_pilon
TLR14_LACH	ENSLACT00000020278	Ensembl v78
TLR14_LEOC	ENSLOCT00000009719	Ensembl v78
TLR14_ORLA	ENSORLT00000019664	Ensembl v67

TLR14_ORNI	ENSONIT00000008447	Ensembl v67
TLR14_PEMA	ENSPMAT00000002031 partial end of scaffold	Ensembl v67
TLR14_POFO	ENSPFOT00000018436	Ensembl v78
TLR14_TARU	ENSTRUT00000001047	Ensembl v67
TLR14_TENI	ENSTNIT00000009097 Partial TIR in gap	Ensembl v67
TLR14_XIMA	ENSXMAT00000013405	Ensembl v78
TLR14a_XETR	ENSXETT00000054703	Ensembl v67
TLR14b_XETR	ENSXETT00000038393	Ensembl v67
TLR14c_XETR	ENSXETT00000054700	Ensembl v67
TLR14d_XETR	ENSXETT00000054707	Ensembl v67
TLR15		
TLR15_ANCA	ENSACAT00000024923 + 3000 bp 5' leader in gap	Ensembl v78
TLR15_GAGA	ENSGALT00000013275	Ensembl v67
TLR21		
TLR21a_GAAC	ENSGACT00000012366	Ensembl v67
TLR21a_LACH	ENSLACT00000001213	Ensembl v78
TLR21_ANCA	ENSACAT00000015088	Ensembl v67
TLR21_ASME	ENSAMXT00000025862	Ensembl v78
TLR21b_GAAC	ENSGACT00000011120	Ensembl v67
TLR21b_LACH	ENSLACT00000002997	Ensembl v78
TLR21_DARE	ENSDART00000060142	Ensembl v67
TLR21_GAGA	ENSGALT00000001120	Ensembl v67
TLR21_GAMO	Contig175	Celera140828_pbjelly_pilon
TLR21_ORLA	ENSORLT00000016853	Ensembl v67
TLR21_ORNI	ENSONIT00000025851	Ensembl v67
TLR21_POFO	ENSPFOT00000020466	Ensembl v78
TLR21_TARU	ENSTRUT00000009778	Ensembl v67
TLR21_TENI	8:1034243:1037340	Ensembl v78
TLR21_XETR	ENSXETT00000061166	Ensembl v67
TLR21a_PEMA	ENSPMAT00000011126	Ensembl v67
TLR21b_PEMA	ENSPMAT00000011208	Ensembl v67
TLR21c_PEMA	ENSPMAT00000005511	Ensembl v67
TLR21_XIMA	ENSXMAT00000010804 partial	Ensembl v78
TLR22		
TLR22a_GAMO	Contig1071	Celera140828_pbjelly_pilon
TLR22b_GAMO	Contig1071	Celera140828_pbjelly_pilon
TLR22c_GAMO	Contig282	Celera140828_pbjelly_pilon
TLR22d_GAMO	Contig7090	Celera140828_pbjelly_pilon
TLR22e_GAMO	Contig282	Celera140828_pbjelly_pilon
TLR22f_GAMO	Contig180	Celera140828_pbjelly_pilon
TLR22g_GAMO	Contig661	Celera140828_pbjelly_pilon
TLR22_GAAC	ENSGACT00000007216	Ensembl v67
TLR22h_GAMO	Contig661	Celera140828_pbjelly_pilon
TLR22i_GAMO	Contig1563	Celera140828_pbjelly_pilon

TLR22j_GAMO	Contig9248	Celera140828_pbjelly_pilon
TLR22k_GAMO	Contig7090	Celera140828_pbjelly_pilon
TLR22l_GAMO	Contig282	Celera140828_pbjelly_pilon
TLR22_ORLA	ENSORLT00000025295	Ensembl v67
TLR22_ORNI	ENSONIT00000008197	Ensembl v67
TLR22_POFO	ENSPFOT00000004640	Ensembl v78
TLR22_TARU	ENSTRUT00000015141	Ensembl v67
TLR22_TENI	ENSTNIT00000016840	Ensembl v67
TLR22a_XIMA	JH557120.1:130052-135644	Ensembl v78
TLR22b_XIMA	JH557120.1:121404-127621	Ensembl v78
TLR23		
TLR23a_ASME	ENSAMXT00000025783	Ensembl v78
TLR23a_ORNI	ENSONIT00000014707	Ensembl v67
TLR23a_POFO	ENSPFOT00000027239	Ensembl v78
TLR23_ANCA	ENSACAT00000023214	Ensembl v67
TLR23b_ASME	ENSAMXT00000026733	Ensembl v78
TLR23b_ORNI	ENSONIT00000004968	Ensembl v67
TLR23b_POFO	ENSPFOT00000031308	Ensembl v78
TLR23c_ASME	ENSAMXT00000026734	Ensembl v78
TLR23c_POFO	ENSPFOT00000011105	Ensembl v78
TLR23_LEOC	ENSLOCT00000022458	Ensembl v78
TLR23_TARU	ENSTRUT00000010682	Ensembl v67
TLR23_TENI	ENSTNIT00000008231	Ensembl v67
TLR23_XETR	ENSXETT00000055333	Ensembl v67
TLR23_GAMO	Contig4895	Celera140828_pbjelly_pilon
TLR23_XIMA	JH556800.1:603094-607350	Ensembl v78
TLR25		
TLR25a_LEOC	ENSLOCT00000020915	Ensembl v78
TLR25b_LEOC	ENSLOCT00000022053	Ensembl v78
TLR25_ICPU	HQ677726.1	GenBank 2013_08
TLR25_ORLA	ENSORLT00000015995	Ensembl v67
TLR25_ORNI	ENSONIT00000017803	Ensembl v67
TLR25a_GAMO	Contig177	Celera140828_pbjelly_pilon
TLR25a_PEMA	ENSPMAT00000011112	Ensembl v67
TLR25b_GAMO	Contig177	Celera140828_pbjelly_pilon
TLR25b_PEMA	ENSPMAT00000011257	Ensembl v67
TLR25c_GAMO	Contig4860	Celera140828_pbjelly_pilon
TLR25d_GAMO	Contig4860	Celera140828_pbjelly_pilon
TLR25e_GAMO	Contig4860	Celera140828_pbjelly_pilon
TLR25f_GAMO	Contig2404	Celera140828_pbjelly_pilon
TLR25g_GAMO	Contig4860	Celera140828_pbjelly_pilon
TLR26		
TLR26_ASME	ENSAMXT00000026984	Ensembl v78
TLR26_ICPU	AEI59681.1 (GenBank)	GenBank 2013_08
TLR26_XETR	ENSXETT00000060548	Ensembl v67
TLR26a_DARE	ENSDART00000124265	Ensembl v67

TLR26b_DARE	ENSDART00000132944	Ensembl v67

Supplementary table 3 – Species overview with abbreviations, common names and latin names

Abbreviation	Common name	Latin name
BRBE	Amphioxus	*Branchiostoma belcheri*
ANCA	Anole lizzard	*Anolis carolinensis*
ASME	Cave fish	*Astyanax mexicanus*
CTEID	Grass carp	*Ctenopharyngodon idella*
CRIMI	Gray dwarf hamster	*Cricetulus migratorius*
CYPCA	Common carp	*Cyprinus carpio*
DARE	Zebrafish	*Danio rerio*
GAAC	Stickleback	*Gasterosteus aculeatus*
GAGA	Chicken	*Gallus gallus*
GAMO	Atlantic cod	*Gadus morhua*
HOSA	Human	*Homo sapiens*
ICPU	Channel catfish	*Ictalurus punctatus*
LACH	Coelacanth	*Latimeria chalumnae*
LEOC	Spotted gar	*Lepisosteus oculatus*
MUMU	Mouse	*Mus musculus*
ONMY	Rainbow trout	*Oncorhynchus mykiss*
ORLA	Medaka	*Oryzias latipes*
ORNI	Tilapia	*Oreochromis niloticus*
PEMA	Lamprey	*Petromyzon marinus*
POFO	Amazon molly	*Poecilia formosa*
RANO	Rat	*Rattus norvegicus*
SASA	Salmon	*Salmo salar*
TARU	Fugu	*Takifugu rubripes*
TENI	Tetraodon	*Tetraodon nigroviridis*
XETR	Xenopus	*Xenopus tropicalis*
XIMA	Platyfish	*Xiphophorus maculatus*

Supplementary table 4 – Reasons for excluding some *TLR* genes found in the Atlantic cod genome from further analysis

TLR gene	Exclusion comment
TLR7c	Significant amount of indels and low read count
TLR8b	Missing leader and two larger deletions
TLR8g	Large deletion and low read count
TLR8h	Significant amount of indels and low read count
TLR8i	Significant amount of indels
TLR8k	Several stop codons and low read count
TLR22i	Significant amount of indels
TLR22k	Significant amount of indels
TLR25b	Significant amount of indels and low read count
TLR25g	Significant amount of indels and low read count

Supplementary table 5 – Protein queries for mapping of the TLR signaling pathway in Atlantic cod

Gene	Identifier
AKT1	ENSGMOP00000012247

AKT1	ENSTRUP00000034366
AKT1	ENSP00000270202
AKT1	ENSGACP00000008334
AP-1	ENSGMOP00000011814
AP-1	ENSGMOP00000016486
AP-1	ENSGMOP00000011824
AP-1	ENSTRUP00000026703
AP-1	ENSGACP00000013845
AP-1	ENSGACP00000010100
AP-1	ENSGACP00000013876
AP-1	ENSP00000306245
CASP8	ENSGMOP00000001325
CASP8	ENSTRUP00000024465
CASP8	ENSP00000351273
CCL3	ENSP00000225245
CCL3	ENSMUSP00000001008
CCL4	ENSP00000250151
CCL4	ENSMUSP00000019074
CCL5	ENSTTRP00000005524
CCL5	ENSP00000293272
CCL5	ENSMUSP00000039600
CD40	ENSGMOP00000004266
CD40	ENSTRUP00000019665
CD40	ENSP00000361350
CD80	ENSP00000264246
CD80	ENSACAP00000020167
CD80	ENSTTRP00000013645
CD80	ENSMUSP00000097404
CD80	ENSP00000264246
CD80	ENSMUSP00000097404
CD80/86	B5M0C2
CD86	ENSTTRP00000010925
CD86	ENSP00000332049
CD86	ENSMUSP00000087047
CD86	ENSP00000332049
CD86	ENSMUSP00000087047
CTSK	ENSGMOP00000005371
CTSK	ENSTRUP00000001247
CTSK	ENSP00000271651
CXCL10	ENSP00000305651
CXCL10	ENSMUSP00000047646
CXCL10	Q7T0B3
CXCL11	ENSTTRP00000008519
CXCL11	ENSP00000306884
CXCL11	ENSDARP00000115162
CXCL11	ENSP00000306884

CXCL11	ENSDARP00000116772
CXCL9	ENSTTRP00000011825
CXCL9	ENSP00000354901
CXCL9	ENSMUSP00000108716
CXCL9	ENSP00000354901
CXCL9	ENSMUSP00000108716
FADD	ENSGMOP00000016954
FADD	ENSP00000301838
FADD	ENSDARP00000089060
FOS	ENSGMOP00000011814
FOS	ENSTRUP00000026703
FOS	ENSP00000306245
IFNA1	ENSP00000276927
IFNA1	ENSMUSP00000092580
IFNA1	Q6XQG7
IFNAB1	B7SVJ8
IFNAR	ENSP00000270139
IFNAR	ENSGMOP00000012014
IFNAR	ENSTRUP00000045127
IFNB1	ENSP00000369581
IFNB1	ENSMUSP00000056720
IkBa	ENSGMOP00000009960
IkBa	ENSGMOP00000018701
IkBa	ENSTRUP00000011820
IkBa	ENSTRUP00000012183
IkBa	ENSP00000216797
IKBKA	ENSGMOP00000003286
IKBKA	ENSGMOP00000006411
IKBKA	ENSTRUP00000022821
IKBKA	ENSTRUP00000036336
IKBKA	ENSP00000359424
IKBKB	ENSGMOP00000012404
IKBKB	ENSTRUP00000041917
IKBKB	ENSP00000404920
IKBKG	ENSGMOP00000006581
IKBKG	ENSTRUP00000005582
IKBKG	ENSP00000358614
IL12	P29459
IL12	P43431
IL12	P29460
IL12	P43432
IL12	B5XFE3
IL12	Q2PD23
IL12	Q2PDJ1
IL12	Q800F2
IL12	Q5DVP6

IL12	Q6UAL9
IL12	Q6UAL8
IL12A	ENSP00000303231
IL12A	ENSMUSP00000029345
IL12A	ENSTTRP00000008806
IL12B	ENSP00000231228
IL12B	ENSMUSP00000099860
IL1B	ENSGMOP00000000339
IL1B	ENSTRUP00000040919
IL1B	ENSP00000263341
IL6	ENSTRUP00000021935
IL6	ENSP00000258743
IL6	ENSXETP00000016123
IL8	ENSP00000306512
IL8	ENSORLP00000006429
IL8	ENSACAP00000011122
IRAK1	ENSTRUP00000016336
IRAK1	ENSP00000358991
IRAK1	ENSGACP00000001480
IRAK4	ENSGMOP00000009738
IRAK4	ENSTRUP00000042842
IRAK4	ENSP00000390651
IRAK4	ENSGACP00000005317
IRF3	ENSGMOP00000013712
IRF3	ENSTRUP00000030442
IRF3	ENSP00000310127
IRF5	ENSGMOP00000007757
IRF5	ENSTRUP00000001526
IRF5	ENSP00000249375
IRF7	ENSGMOP00000010233
IRF7	ENSTRUP00000028834
IRF7	ENSP00000329411
JNK	ENSGMOP00000005758
JNK	ENSTRUP00000032787
JNK	ENSTRUP00000043883
JNK	ENSP00000363304
JNK	ENSGACP00000009405
JNK	ENSGACP00000007559
LBP	ENSP00000217407
LBP	ENSMUSP00000016168
LY96	ENSTTRP00000012874
LY96	ENSP00000284818
LY96	ENSMUSP00000026881
MD-2	ENSP00000284818
MD-2	ENSMUSP00000026881
MYD88	ENSGMOP00000011696

MYD88	ENSTRUP00000044800
MYD88	ENSGACP00000004643
MYD88	ENSP00000379625
NFKB1	ENSGMOP00000008036
NFKB1	ENSP00000226574
NFKB1	ENSGACP00000022290
p38	ENSP00000229795
p38	ENSDARP00000040361
p38	ENSDARP00000035686
p38	ENSMUSP00000004990
PI3KCA	ENSTRUP00000016050
PI3KCA	ENSTRUP00000009530
PI3KCA	ENSP00000263967
PI3KCA	ENSGACP00000021206
PI3KCA	ENSGACP00000008159
PI3KCA	ENSGMOP00000003184
PI3KCA	ENSGMOP00000009146
Rac1	ENSGMOP00000009517
Rac1	ENSP00000258737
Rac1	ENSORLP00000010995
RANTES	ENSP00000293272
RANTES	ENSMUSP00000039600
RIP1	ENSGMOP00000019617
RIP1	ENSTRUP00000029781
RIP1	ENSGACP00000017178
STAT1	ENSGMOP00000013310
STAT1	ENSTRUP00000000221
STAT1	ENSP00000354394
TAB1	ENSTRUP00000000047
TAB1	ENSP00000333049
TAB1	ENSDARP00000053182
TAB2	ENSGMOP00000015412
TAB2	ENSTRUP00000019226
TAB2	ENSP00000445752
TAK1	ENSGMOP00000018726
TAK1	ENSGMOP00000002024
TAK1	ENSTRUP00000014299
TAK1	ENSP00000358335
TIRAP	ENSP00000376446
TIRAP	ENSGACP00000008666
TIRAP	ENSDARP00000103037
TNF	ENSP00000389265
TNF	ENSP00000365290
TNF	ENSDARP00000025726
TNF	ENSXETP00000005341
TNF	ENSMUSP00000025263

TNFA	ENSP00000372988
TNFA	ENSDARP00000025726
TNFA	ENSTRUP00000014387
TOLLIP	ENSGMOP00000011676
TOLLIP	ENSP00000314733
TOLLIP	ENSTRUP00000001350
Tpl2	ENSGMOP00000002622
Tpl2	ENSP00000443610
Tpl2	ENSTRUP00000007084
TRAF3	ENSGMOP00000015237
TRAF3	ENSTRUP00000013348
TRAF3	ENSP00000454207
TRAF6	ENSGMOP00000015220
TRAF6	ENSTRUP00000041697
TRAF6	ENSP00000433623
TRAM	ENSP00000415139
TRAM	Q8BJQ4
TRIF	ENSTNIP00000003841
TRIF	ENSP00000248244
TRIF	ENSDARP00000106233
TRIF	ENSDARP00000062684

Supplementary table 6 – Atlantic cod *IL8* copies with contig location and abbreviations

Assembly scaffold	Abbreviation
Contig1401_2	IL8a
Contig1401_rev	IL8b
Contig1401_1	IL8c
Contig1592_maybegap	IL8d
Contig1411_noleader	IL8e
Contig1549_rev	IL8f
Contig1411_1	IL8g
Contig1864_rev	IL8h

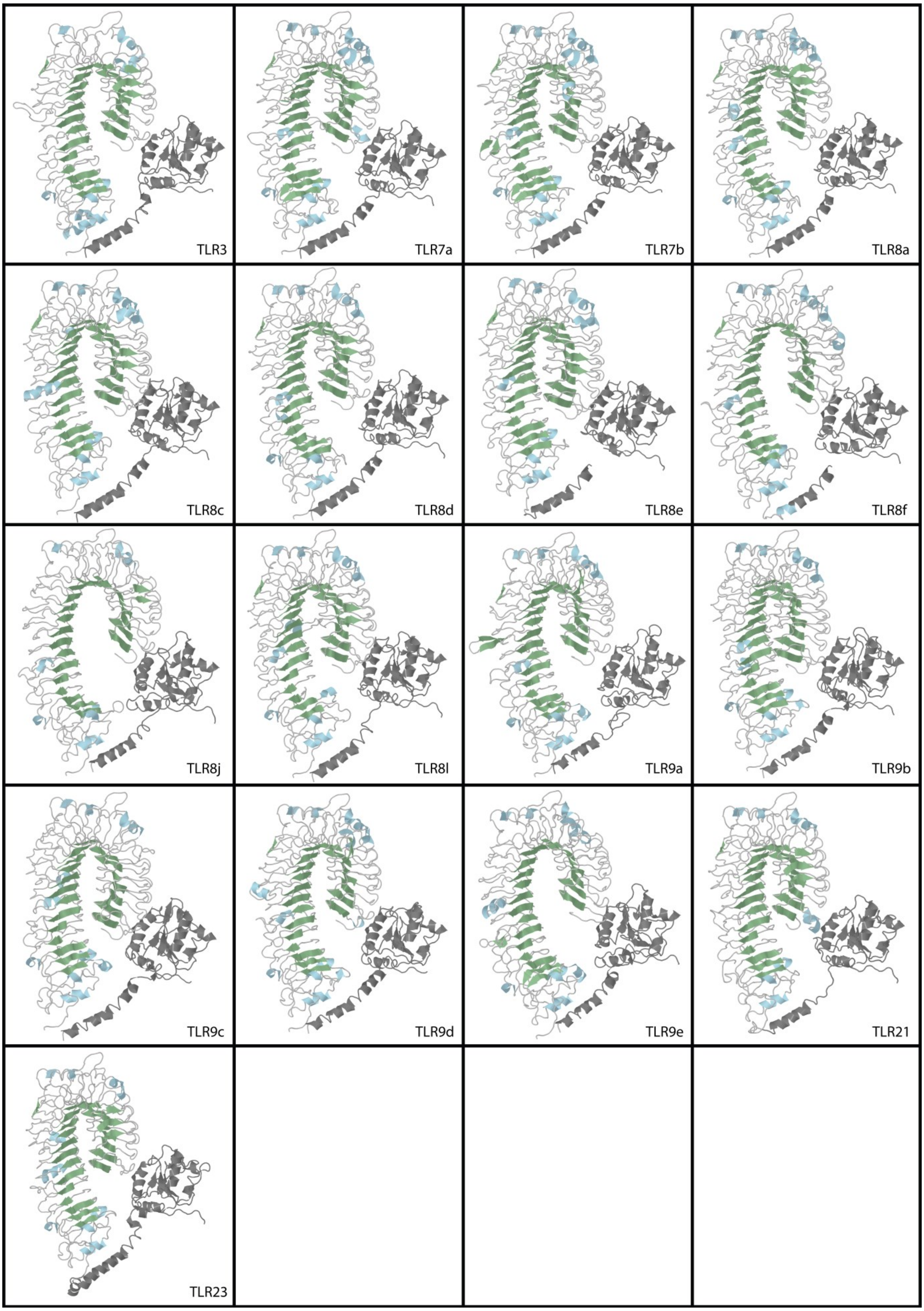

Supplementary figure 1 – Protein structure models of likely intracellularly located TLRs in Atlantic. All are modelled onto the human TLR5 crystal structure. The transmembrane, linker and TIR domain is colored dark grey whereas the ecto-domain is colored light grey with its sheets in pale green and

helices in light blue. Sheets overlap with leucine-rich repeats in the ecto-domain.These TLRs display longer solenoid structures due to a larger number of leucine-rich repeat elements.

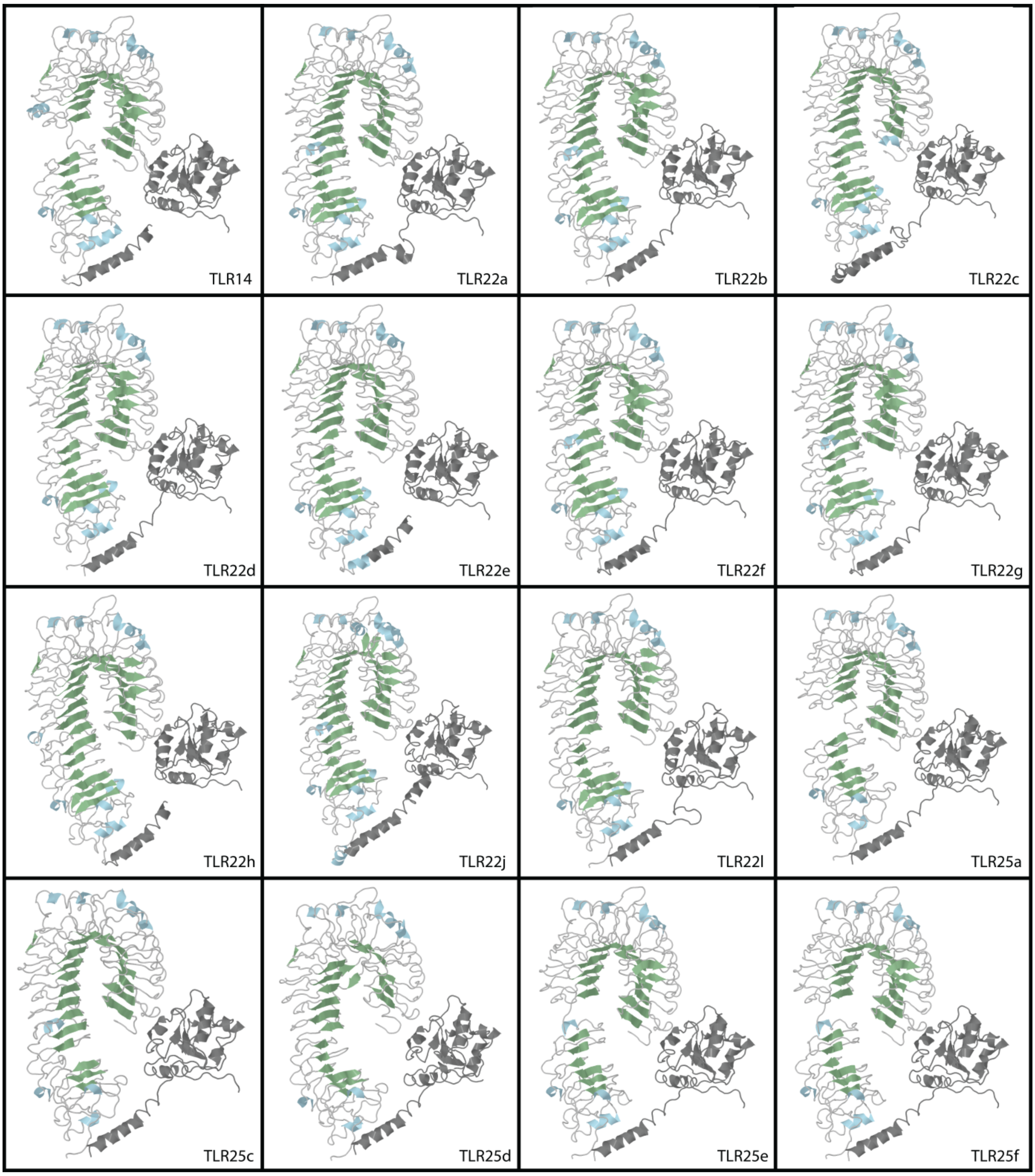

Supplementary figure 2 - Protein structure models of likely plasma membrane associated TLRs in Atlantic. All are modelled onto the human TLR5 crystal structure. The transmembrane, linker and TIR domain is colored dark grey whereas the ecto-domain is colored light grey with its sheets in pale green and helices in light blue. Sheets overlap with leucine-rich repeats in the ecto-domain.These TLRs display shorter solenoid structures with the exception of TLR22(see references Matsuo, et al. 2008; Reyes-Becerril, et al. 2015; Salazar, et al. 2015 for more details on putative TLR22 function).

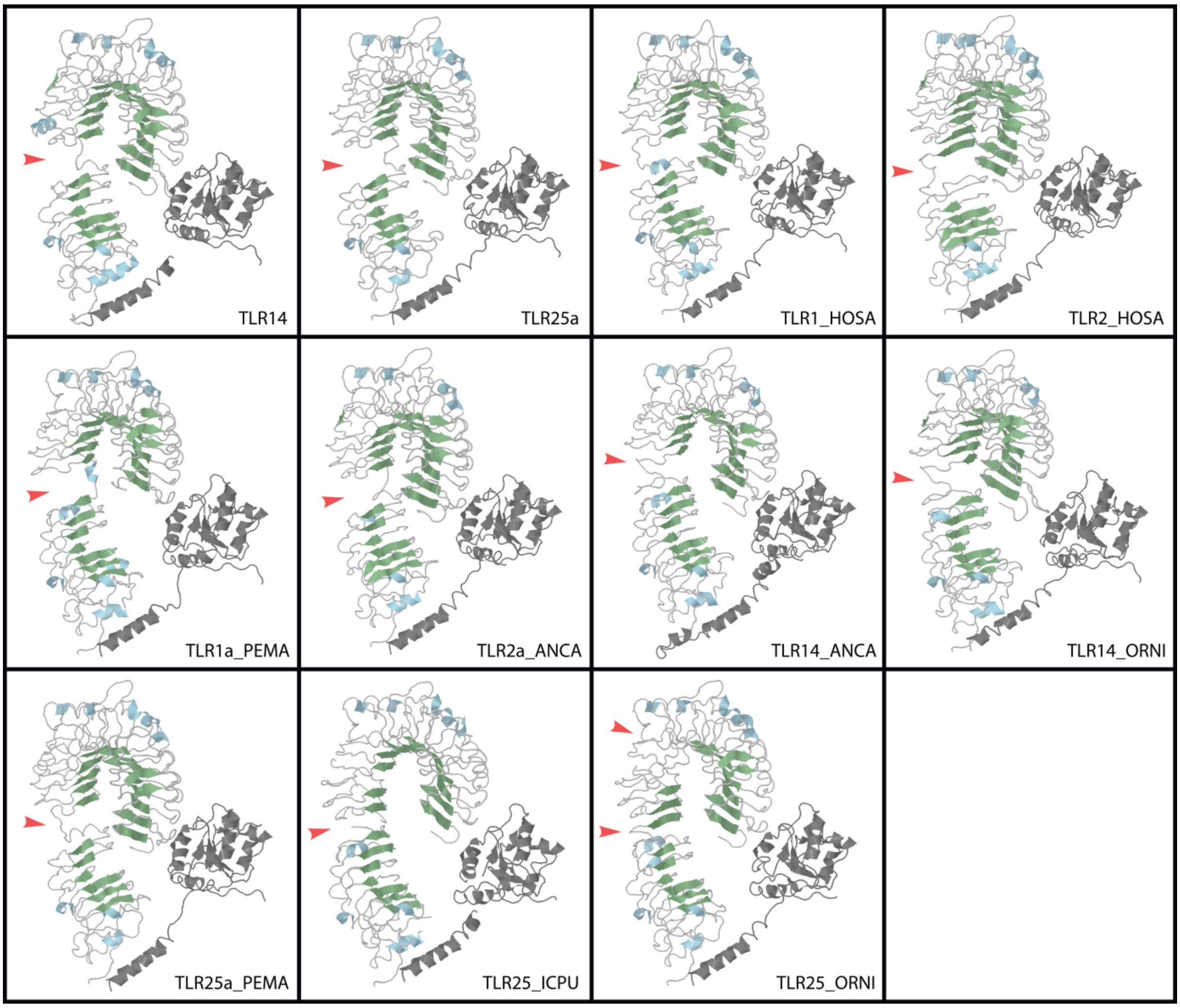

Supplementary figure 3 – A comparison of protein structure models of *TLR1*-family TLRs in Atlantic cod, lamprey, anole lizard, tilapia and channel catfish. All are modelled onto the human TLR5 crystal structure. The transmembrane, linker and TIR domain is colored dark grey whereas the ecto-domain is colored light grey with its sheets in pale green and helices in light blue. Sheets overlap with leucine-rich repeats in the ecto-domain. The shorter solenoid structre reveals itself by longer loops being introduced into the predicted structure – here marked by red arrows.

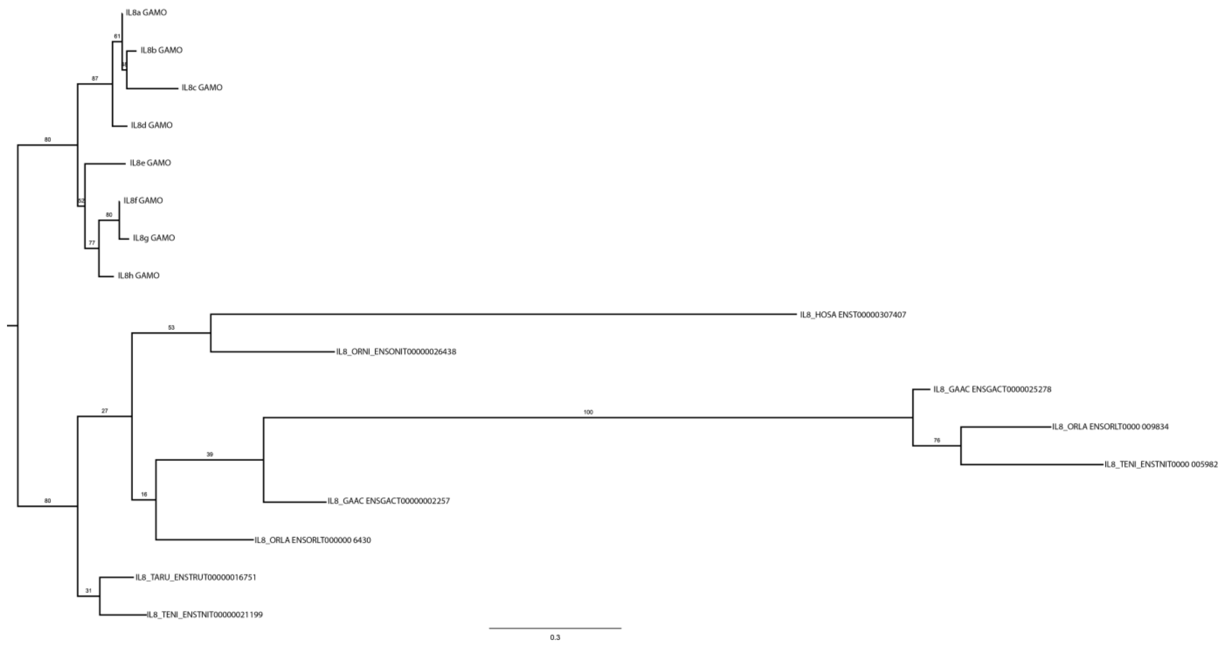

Supplementary figure 4 – The Atlantic cod *IL8* copies together with *IL8* from fugu, tetraodon, medaka, tilapia, stickleback and human in a full length protein sequence ML-phylogeny with bootstrap values. Atlantic cod *IL8s* form two clades and we also found *IL8* duplications in some of the investigated fish species.
